# Gene Therapy for Malignant and Benign Gynaecological Disorders: A Systematic Review of an Emerging Success Story

**DOI:** 10.3390/cancers14133238

**Published:** 2022-06-30

**Authors:** Ekati Drakopoulou, Nicholas P. Anagnou, Kalliopi I. Pappa

**Affiliations:** 1 Laboratory of Cell and Gene Therapy, Biomedical Research Foundation of the Academy of Athens (BRFAA), 11527 Athens, Greece; edrakopoulou@bioacademy.gr (E.D.); kpappa@med.uoa.gr (K.I.P.); 2 First Department of Obstetrics and Gynecology, University of Athens School of Medicine, 11528 Athens, Greece

**Keywords:** gene therapy, gynaecological cancer, ovarian cancer, cervical cancer, viral vectors, non-malignant gynaecological disorders

## Abstract

**Simple Summary:**

This review discusses all the major advances in gene therapy of gynaecological disorders, highlighting the novel and potentially therapeutic perspectives associated with such an approach. It specifically focuses on the gene therapy strategies against major gynaecological malignant disorders, such as ovarian, cervical, and endometrial cancer, as well as benign disorders, such as uterine leiomyomas, endometriosis, placental, and embryo implantation disorders. The above therapeutic strategies, which employ both viral and non-viral systems for mutation compensation, suicide gene therapy, oncolytic virotherapy, antiangiogenesis and immunopotentiation approaches, have yielded promising results over the last decade, setting the grounds for successful clinical trials.

**Abstract:**

Despite the major advances in screening and therapeutic approaches, gynaecological malignancies still present as a leading cause of death among women of reproductive age. Cervical cancer, although largely preventable through vaccination and regular screening, remains the fourth most common and most lethal cancer type in women, while the available treatment schemes still pose a fertility threat. Ovarian cancer is associated with high morbidity rates, primarily due to lack of symptoms and high relapse rates following treatment, whereas endometrial cancer, although usually curable by surgery, it still represents a therapeutic problem. On the other hand, benign abnormalities, such as fibroids, endometriosis, placental, and embryo implantation disorders, although not life-threatening, significantly affect women’s life and fertility and have high socio-economic impacts. In the last decade, targeted gene therapy approaches toward both malignant and benign gynaecological abnormalities have led to promising results, setting the ground for successful clinical trials. The above therapeutic strategies employ both viral and non-viral systems for mutation compensation, suicide gene therapy, oncolytic virotherapy, antiangiogenesis and immunopotentiation. This review discusses all the major advances in gene therapy of gynaecological disorders and highlights the novel and potentially therapeutic perspectives associated with such an approach.

## 1. Introduction

**Figure 1:**
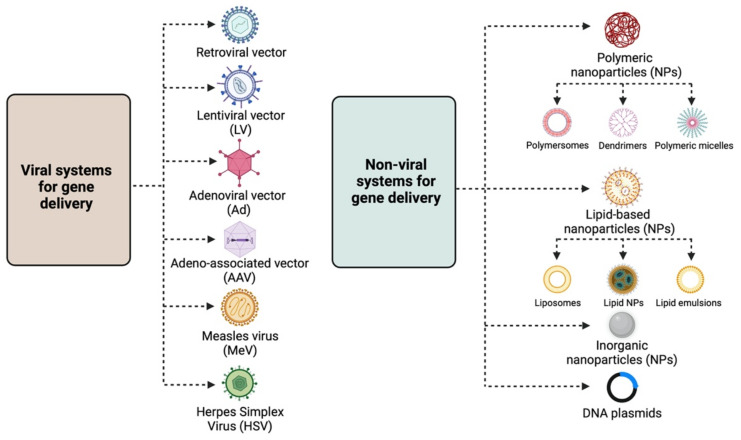
Viral and non-viral systems for gene delivery.

The continuously growing amount of information regarding the molecular mechanisms underlying the emergence of tissue abnormalities from benign tumours to invasive malignancies has set the grounds for the use of gene therapy as a rational yet radical therapeutic approach for cancer therapy [1]. Gene therapy aims to introduce or modify genetic material into target cells, thus altering their function, usually by either restoring a lost function or initiating a new one. Although it was initially employed for the treatment of inherited genetic diseases, gene therapy was soon identified as an effective approach for the treatment of both gynaecological malignancies such as ovarian [2], cervical [3], and endometrial cancer [4] and certain benign gynaecological abnormalities, such as leiomyomas, endometriosis, placental, and embryo implantation disorders [5]. There are two main strategies for specific and efficient gene delivery to cancer and non-cancer cells, and these involve either viral or non-viral systems, as summarised in Figure 1 and described below in Section 2.

**Figure 2:**
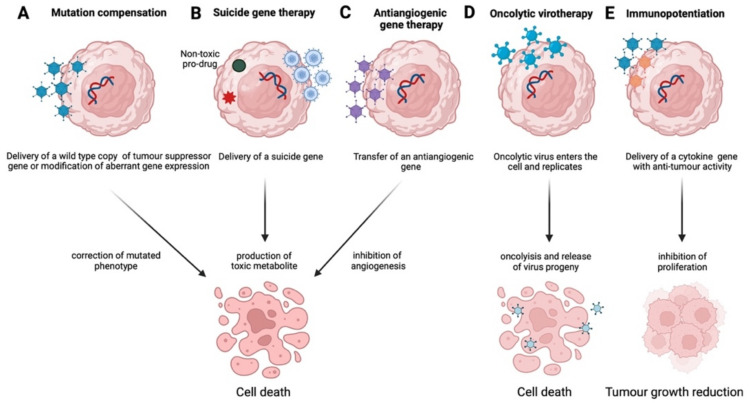
Main targeted approaches for the gene therapy of gynaecological malignant and benign disorders. They mainly involve (**A**) Mutation compensation employing primarily adenoviral vectors to correct the mutated phenotype or modify aberrant gene expression in tumour cells, usually leading to cancer cell death (**B**) Suicide gene therapy, usually with the delivery of a suicide gene, such as *HSV-TK*, leading to cell death following the production of toxic metabolite (**C**) Antiangiogenic gene therapy, by transferring an antiangiogenic gene, which inhibits cancer cell angiogenesis resulting in cancer cell death (**D**) Oncolytic virotherapy using oncolytic viruses that enter the cell, replicate and lead to oncolysis and cancer cell death. (**E**) Immunopotentiation by delivering a potent anti-cancer cytokine gene within the tumour using primarily adenoviruses and adeno-associated viral vectors, leading to reduction in tumour growth.

The main gene therapy strategies employ mutation compensation, primarily involving the replacement of a mutated tumour suppressor gene or the modification of aberrant gene expression in tumour cells, antiangiogenic, suicide gene therapy, and immunopotentiation approaches [5,6] (Figure 2). This review covers all major advances in gene therapy for gynaecological cancer and benign disorders.

## 2. Gene Delivery Systems

The main strategies for targeted gene therapy approaches for malignant and benign gynaecological disorders employ both viral and non-viral systems (Figure 1). This section provides a brief overview of the different viral and non-viral gene delivery systems.

### 2.1. Viral Systems

1. Retroviral vectors: Lentiviral vectors (LVs) are the most widely used retroviruses for therapeutic gene delivery, as they can efficiently transduce both quiescent and dividing cells and thus facilitate safe, efficient, and stable transgene expression [7,8]. They are enveloped, single-stranded RNA viruses with a packaging capacity of ~9 kb [7] and have already been employed in numerous successful clinical trials for the treatment of various diseases, such as hemoglobinopathies, metabolic and immune disorders, and various cancer types, including gynaecological malignancies [9]. Moreover, they have been instrumental in the development of chimeric antigen receptor (CAR) T cells, an approach with promising therapeutic perspectives in the field of oncology;
2. Adenoviral vectors (Ads): Adenoviral vectors have been extensively used as viral vector platforms primarily due to their broad tropism and the high transduction efficiency of both quiescent and dividing cells, as well as for their capacity to persist as episomal elements within the target cells [9]. They mainly derive from human serotypes-2 (Ad2) and -5 (Ad5) adenoviruses, which are non-enveloped, double-stranded DNA viruses with an icosahedral capsid able to accommodate up to 45 kb linear, double-stranded DNA [10]. However, since they are usually associated with potent immune responses [11] due to pre-existing immunity, extensive research has focused on generating less immunogenic Ad vectors, employing serotypes with low seroprevalence, such as Ad26 and Ad35 [12]. Furthermore, the generation of conditionally replicative adenoviral vectors (CRAds) by introducing tumour-specific promoters into the Ad genome can lead to higher gene expression [13]. Regarding gene therapy for malignant and benign gynaecological disorders, Ads have been extensively used as therapeutic vectors in both pre-clinical and clinical studies [9] for the delivery of vaccines, tumour suppressor genes, suicide genes, and immunomodulatory genes. All of these approaches are discussed in detail below;
3. Adeno-associated vectors (AAVs): Adeno-associated vectors are single-stranded DNA viruses with an icosahedral capsid of ~5 kb packaging capacity that depend on adenoviruses to complete their life cycle. The current AAV1 and AAV2 serotypes used are characterised by broad tropism, stable episomal persistence, and reduced immunogenicity, and therefore, they have been widely employed in gene therapy approaches [9]. Although AAV vectors have not been used yet in clinical gene therapy trials for gynaecological disorders, encouraging data from their pre-clinical assessment presented in this review point toward this direction;
4. Measles virus (MeV): The vaccine strain of MeV is a negative-strand RNA virus with a 16 kb-long genome that presents an attractive oncolytic platform, mainly due to its ability to selectively infect malignant cells, which overexpress the CD46 receptor [14]. Due to the fact that the monotherapy oncolytic approach using MeV is usually not sufficient to treat advanced-stage malignancies, combination approaches using radiotherapy or chemotherapy, MeV harbouring with therapeutic or immunomodulatory genes, and the use of carrier cells for MeV delivery into tumour cells can lead to successful therapeutic outcomes for a number of malignancies, including gynaecological cancers [15];
5. Herpes Simplex Virus (HSV): HSV-1 and HSV-2 members are enveloped, double-stranded DNA viruses with an icosahedral capsid, which demonstrate attractive vector features, such as a large accommodation capacity, easy production, and high titers [16]. Apart from their ability to mediate oncolysis upon delivery of the suicide thymidine kinase *(TK)* suicide gene into malignant cells, HSV vectors have also been used to deliver immunomodulatory cytokines into tumour cells and thus elicit a strong anti-tumour immune response. Extensive research has focused on the development of safer HSV vectors, carrying transcriptionally active promoter elements that overcome the latency of the viral genome, aiming to expand HSV pre-clinical and clinical applications [16].

### 2.2. Non-Viral Systems

The need for non-viral gene delivery systems arose primarily due to the immunogenicity and cytotoxicity concerns raised by some viral vectors, such as Adenoviruses [11]. Despite the relatively low transfection efficiency, their use in the field of gene therapy has gained a lot of ground [13,17]. The main non-viral approaches involve the delivery of the desired gene material, e.g., naked or plasmid DNA, either by physical methods, such as electroporation, ballistic DNA, injection, photoporation, magnetofection, sonoporation, and hydroporation [13,18], or chemically, by means of polymeric, lipid-based and inorganic nanoparticles (NPs) [13,19]. 

#### 2.2.1. Physical Methods

Briefly, the most common types of non-viral physical methods involve the following approaches.

1. Electroporation: The electric pulse creates pores in the cell membrane, allowing the genetic material to enter the target cell. Depending on the target tissue, the electric pulse differs both in strength (high or low) and duration (short or long pulses), with cancer cells usually requiring low field strength (<700 V/cm) with long pulses (milliseconds) [13];
2. Ballistic DNA: Bombardment of DNA-coated heavy metal particles into target cells at a certain speed. The precision in DNA delivery makes it a method of choice for ovarian cancer [20];
3. Injection: The genetic material is directly injected into tissue by means of a needle. It represents the preferred approach for solid tumours; however, it has a relatively low efficiency [20];
4. Photoporation: A laser pulse causes cells to be permeable to DNA, with transfection efficiency being dependent on the focal point and laser frequency [13];
5. Magnetofection: It is mostly used for in vitro approaches; a gene-magnetic particle complex is introduced in cell culture, and electromagnets placed below the cell culture generate magnetic fields that mediate its effective sedimentation, thus leading to higher transfection efficiency [13];
6. Sonoporation: The generated ultrasound waves cause cell membranes to be permeable to micro-bubbles containing gene products [13];
7. Hydroporation: Hydrodynamic pressure generated by the injection of a large volume of DNA in a short period of time increases cell membrane permeability and mediates gene delivery [13].

#### 2.2.2. Chemical Vectors 

Non-viral chemical delivery systems employ particles of different shapes and sizes, known as nanoparticles (NPs), to encapsulate and efficiently transport genetic material, such as DNA and RNA, into target cells via endocytosis. These NPs are divided into the following three classes [13,19].

1. Polymeric NPs: These are stable, biodegradable, and water-soluble NPs, which make ideal candidates for drug delivery. They exist as monomers or polymers of different structures, with the most common being nanocapsules and nanospheres. According to shape, they are further divided into polymersomes, dendrimers, and micelles. Polymersomes are artificial vesicles with amphiphilic membranes that display increased cargo capacity and effective cytosol delivery [21], whereas micelles are quite effective for aqueous drug administration due to their hydrophilic core and hydrophobic coating. Polymeric micelles have already been employed in clinical trials, specifically in a study testing the effective delivery of paclitaxel (PTX), a drug widely used for ovarian cancer treatment [22]. Dendrimers are more complex, three-dimensional nanocarriers, commonly used for the delivery of nucleic acids and drugs in cancer therapy, usually in combination with poly amidoamine (PAMAM) and polyethylenimine (PEI) polymers [23]. Despite the toxicity associated with the surface amino groups, PAMAM was the first dendrimer used for gene delivery [17];
2. Lipid-based NPs: They are usually spherical vehicles with an internal aqueous compartment and an external lipid bilayer. They are divided into liposomes or lipoplexes, lipid emulsions (or nanogels), and lipid NPs [17]. Due to their positive charge, cationic liposomes bind to the anionic phosphate group of nucleic acids, forming lipoplexes and interacting with cell membranes, and thus, efficiently deliver nucleic acids into the cell [17]. Lipid emulsions, which are composed of oil, water, and a surface-active agent, show increased stability and serum resistance, whereas solid lipid NPs can protect nucleic acids from nucleases and are primarily employed for siRNA delivery [20];
3. Inorganic NPs: These nanoparticles are composed of inorganic materials, such as iron, gold, and silica, and therefore possess unique electrical, magnetic, and optical properties [13,19]. The most widely used inorganic NPs are gold NPs, primarily due to their low toxicity, while their photothermal properties establish them as good candidates for cancer therapy [24].

## 3. Gene Therapy for Gynaecological Cancers

### 3.1. Ovarian Cancer

**Figure 3:**
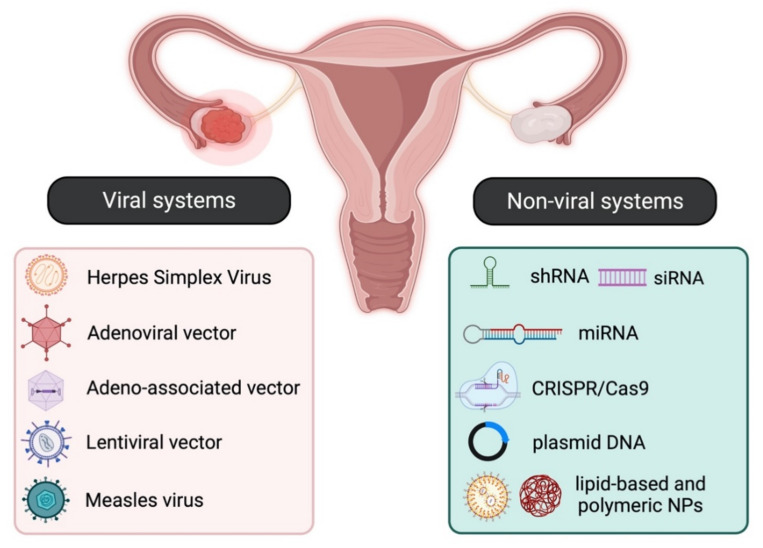
Gene therapy strategies for ovarian cancer. These employ viral vectors, such as Herpes Simplex Virus, adenoviral and adeno-associated viral vectors, lentiviral vectors and Measles virus, and non-viral systems, such as shRNA/siRNA/miRNA, DNA plasmids, lipid-based and polymeric NPs and CRISPR/Cas9 approaches. Of note, some of the above approaches, e.g., shRNA, were also delivered via lentiviral vectors.

Although usually considered a single disease, ovarian cancer is a highly heterogeneous group, comprising different histological subtypes with different underlying molecular mechanisms, clinical manifestations, and hence therapeutic approaches [25]. These include serous, endometrioid, clear-cell, and mucinous carcinoma [26] epithelial ovarian cancer types, which comprise the majority of ovarian cancers. Gene therapy is an emerging therapeutic approach for ovarian cancer [2] and is primarily based on viral and non-viral delivery systems (Figure 3) and on strategies such as suicide and antiangiogenic gene therapy, oncolytic virotherapy, mutation compensation, and immunopotentiation (Table 1).

#### 3.1.1. Viral Vectors

The main viral vectors used for the gene therapy of ovarian cancer are summarized in Table 1 and Figure 3 and include retroviruses (primarily LVs), Ads, AAVs, HSV, and MeV. Highlighted below are the major advancers for each vector type.

**Table 1:** Targeted gene therapy approaches for ovarian cancer.

Gene Therapy Strategy	Gene	Delivery System	Outcome	Model/Species	References
Viral Vectors					
Suicide gene therapy	*HSV-TK*	Adenoviral vector	Enhanced tumour inhibitory effect, increased apoptosis and reduced micro-vessel density	in vitro (human) in vivo (mouse)	[27,28]
Suicide gene therapy	*NTR*	Ad-NTR/Ad-CB1954	Increased survival in ovarian-infected mouse models	in vitro (human) in vivo (mouse)	[29]
Antiangiogenic strategy	*COL18A1* *(endostatin)*	Adenoviral vector Ad-hEndo	Increase in apoptosis, tumour growth and vascularity reduction and prolonged survival	in vitro (mouse/human) in vivo (mouse)	[30,31]
Antiangiogenic strategy	*VEGFR2* *Tie2*	Adenoviral vector Ad-VEGFR2/Ad-Tie2	Significant reduction in tumour weights and accumulation of ascites	in vivo (mouse)	[32]
Antiangiogenic strategy	*P125A-* *COL18A1*	Adeno-associated vector rAAV	Inhibition of tumour growth and blood vessel formation	in vivo (mouse)	[33]
Antiangiogenic strategy	*kringle 5* (K5)	Adeno-associated vector rAAV-K5	Inhibition of VEGF and tumour angiogenesis	in vivo (mouse)	[34]
Antiangiogenic strategy	*VEGF* inhibitor	Adeno-associated vector AAVrh10.BevMab	Significant reduction in tumour growth, reduced angiogenesis, and increased survival in mouse model	in vivo (mouse)	[35]
Mutation compensation	*Bim/Bid*	Adenoviral vector Ad-Bim, Ad-tBid	Strong anti-tumour effect and tumour sensitization in cisplatin chemotherapy following treatment with Ad-tBid	in vitro (human) in vivo (mouse)	[36]
Mutation compensation	*p53*	Adenoviral vector rAd-53	p53-induced apoptosis and taxol sensitization; 90% response rate and 100% peritoneal effusion resolution	in vitro (human) in vivo (human/mouse)	[37,38]
Oncolytic virotherapy	*HER2/neu* *EpCAM*	Measles virus (MeV)	Oncolytic potential	in vitro (human) in vivo (mouse)	[39]
Oncolytic virotherapy /Immunopotentiation	*IL12*	Herpes simplex virus	Cytotoxic effect, reduced metastasis, increased CD8^+^ T-cell response	in vitro (human/mouse) in vivo (mouse)	[40]
Oncolytic virotherapy/ Immunopotentiation	*GM-CSF*	Herpes simplex virus HF10	Reduction in tumour size and increased anti-tumour immune response	in vitro (mouse) in vivo (mouse)	[41]
Oncolytic virotherapy	*Jak1/2* inhibitor	VSP-GP	Transient tumour remission	in vitro (human) in vivo (mouse)	[42]
Oncolytic virotherapy	*STAT3*	Adenoviral vector M4	Inhibition of cell survival and enhanced cisplatin-induced apoptosis	in vitro (human) in vivo (mouse)	[43]
Oncolytic virotherapy	*ZD55-MnSOD*	Adenoviral vector ZD55-MnSOD	Enhanced cisplatin-induced growth suppression and apoptosis	in vitro (human) in vivo (mouse)	[44]
Oncolytic virotherapy	*CD/5FU*	Measles virus MeV-SCD	Effective infection and lysis of cancer cells	in vitro (human)	[45]
Immunopotentiation	*HER2/neu*	Adeno-associated vector rAAV	Strong anti-tumour CTL response	in vitro (human)	[46]
Immunopotentiation	*IL21*	Lentiviral vector pUCMSCs-LV-IL-21	Reduction in tumour size by inhibition of growth, elevation of IFN-γ and TNF-α	in vitro (human) in vivo (mouse)	[47]
Mutation compensation	*CD59*	Retroviral vector shCD59	Enhanced complement-mediated cell damage, increased apoptosis and inhibition of tumour growth	in vitro (human) in vivo (mouse)	[48]
Mutation compensation	*NOB1*	Lentiviral vector shRNA	Suppression of cancer cells growth and colony formation	in vitro (human)	[49]
**Non-viral systems**					
Suicide gene therapy	*hEndoyCD*	Fusion protein in plasmid vector (SV- hEndoyCD)	Inhibition of tumour growth and increased survival in xenograft mouse models	in vitro (human) in vivo (mouse)	[50]
Suicide gene therapy	*gelonin toxin*	HPEI nanogels	Cancer cells growth reduction and induction of apoptosis	in vitro (human) in vivo (mouse)	[51]
Suicide gene therapy	*DT-A*	Cationic polymer	Inhibition of tumour growth and prolonged lifespan in mice	in vitro (human) in vivo (mouse)	[52,53]
Suicide gene therapy	*HSV-tTK*	Tat/pDNA/C16TAB nanoparticle	Increased targeted delivery in mouse model with human ovarian cancer	in vitro human) in vivo (mouse)	[54]
Suicide gene therapy	*VSV-MP*	F-LP/pMP lipoplex	Inhibition of cancer cells proliferation, induction of apoptosis and suppression of tumour angiogenesis	in vitro (human) in vivo (mouse)	[55]
Suicide gene therapy	*CRB1*	PAMAM dendrimer	Increased survival in mice, decreased proliferation, and dissemination of cancer cells	in vitro (human) in vivo (mouse)	[56]
Immunopotentiation	*IL-21*	pIRES2-IL-21-EGFP	Reduction in tumour size, increase in IFN-γ, TNF-α and extension of survival	in vitro (human) in vivo (mouse)	[57]
Immunopotentiation	*pmIL-12*	pmIL-12/PPC	Elevation of IL-12 and IFN-γ, significant decrease in VEGF and survival improvement	in vitro (human) in vivo (mouse)	[58]
Mutation compensation	*hPNAS-4*	cationic liposome	Efficient inhibition of growth, induction of apoptosis, inhibition of angiogenesis	in vitro (human) in vivo (mouse)	[59]
Mutation compensation	*PTEN*	PTEN plasmid	Apoptosis induction, G1 arrest, decrease in migration and invasion	in vitro (human)	[60]
Mutation compensation	*p16/* *eEF1A2*	pcDNA3.1-p16/ pcDNA3.1-vi5-hisA-eEF1A2 plasmids	Inhibition of cell growth by downregulation of eEF1A2	in vitro (human) in vivo (mouse)	[61,62]
Mutation compensation	*WWOX*	recombinant plasmid/ pcDNA3.1-WWOX	Decreased proliferation, cell cycle arrest at G0/G1 phase, induction of apoptosis, inhibition of cell proliferation and autophagy	in vitro (human)	[63,64]
Mutation compensation	*WWOX*	pCMV-WWOX in liposome	Decreased ovarian cancer cell growth and induction of apoptosis	in vitro (human)	[65]
Mutation compensation	*EGFR*	siRNA in nanogels	Chemosensitisation to doxetacel	in vitro (human)	[66]
Mutation compensation	*MACC1*	shRNA	Inhibition of proliferation, migration and invasion, apoptosis induction	in vitro (human)	[67]
Mutation compensation	*MTA1*	siRNA	Reduction in migration, invasion, and adhesion, induction of apoptosis	in vitro (human)	[68]
Mutation compensation	*COX2*	siRNA/shRNA	Inhibition of cell proliferation, cell cycle arrest, reduced invasion and migration, growth suppression	in vitro (human) in vivo (mouse)	[69,70]
Mutation compensation	*WT1*	ASODN in liposomes	Inhibition of cell proliferation, cell cycle arrest, and increased apoptosis	in vitro *(*human)	[71]
Mutation compensation	*STAT3*	shRNA in liposome	Inhibition of cell proliferation and induction of apoptosis	in vitro *(*human) in vivo (mouse)	[72]
Mutation compensation	*NOTCH1*	Cationic-cholesterol derivative-based liposome	Inhibition of growth and apoptosis induction	in vitro *(*human)	[73]
Mutation compensation	*CLDN3*	siRNA in lipidoids shRNA in nanoparticles	Inhibition of tumour growth, reduction in cell proliferation and angiogenesis, apoptosis induction	in vitro (human) in vivo (mouse)	[74,75]
Mutation compensation	*HIF-1a*	siRNA with nanoparticles	Effective inhibition of proliferation	in vitro (human) in vivo (mouse)	[76]
Mutation compensation	*gDNMT1*	F-LP-delivered CRISPR/Cas9	Inhibition of tumour growth	in vitro *(*human) in vivo (mouse)	[77]

##### Retroviruses

In a tumour-suppressor cervical-cancer gene therapy approach, Shi et al. employed shRNA to knock-down CD59, a negative regulator of complement activation, and demonstrated enhanced complement-mediated cell damage, increased apoptosis, and the inhibition of tumour growth in vitro and in vivo [48]. In another study, a lentiviral vector carrying shRNA for Nin one binding protein (NOB1p), a protein frequently overexpressed in ovarian cancer cells, led to the suppression of cancer cell growth and colony formation [49]. Lastly, Zhang et al. transplanted umbilical cord mesenchymal stem cells (MSCs) engineered to express IL-21 in SKOV3 ovarian cancer xenograft-bearing nude mice, utilising lentiviral vector pUCMSCs-LV-IL-21 and demonstrated a reduction in tumour size through the inhibition of growth and the elevation of IFN-γ and TNF-α [47].

##### Adenoviruses (Ads) and Adeno-Associated Viruses (AAVs) 

These viral systems have been widely used in ovarian cancer gene therapy employed in suicide gene therapy, antiangiogenic, and oncolytic virotherapy, alone or together with immunopotentiation, as well as mutation compensation approaches. With regards to suicide gene therapy, Rawlinson et al. [27] developed an adenoviral vector carrying the *HSV-TK* gene under the tumour-specific promoter HE4 and demonstrated increased cell death by prodrug ganciclovir (GCV) treatment in vitro, in ovarian cancer cell lines. When Zhou et al. applied the above system in vivo in a murine ovarian cancer model, they observed an enhanced anti-tumour effect and increased apoptosis, as well as a reduction in micro-vessel density [28]. In a similar approach, but using the NTR-CB1954 system instead, White et al. performed an adenoviral delivery of both *NTR* and CB1954 in ovarian cancer cells and achieved increased survival in both in vitro and in vivo [29]. 

The antiangiogenic approach using Ads and AAVs has also yielded promising results in ovarian cancer, as demonstrated by several studies described in this section. Specifically, Tuppurainen et al. [32] used a combined strategy using adenoviral vectors expressing important genes in angiogenesis, such as vascular endothelial growth factor *(VEGF) 2* receptor and angiopoietin growth factor receptor *Tie2*, with or without chemotherapy, and demonstrated reduced tumour growth and ascites formation. In a different approach, Hampl et al. [30] constructed the adenoviral vector, Ad-hEndo, and used it to mediate the transfer of human endostatin (*COL18A1*) in ovarian cancer ascites in mice, demonstrating a significant down-regulation in ascites formation, reduced tumour growth and vascularity, as well as prolonged animal survival [30]. In order to enhance transfection efficiency and escape antibody neutralisation in vivo, Yang et al. encapsulated the above vector into PEG-PE cationic liposomes and demonstrated the suppression of ovarian cancer growth in vivo, in ovarian cancer mouse models [31]. Similarly, the adeno-associated-mediated delivery of *P125A- COL18A1*, a mutated endostatin gene, led to the inhibition of tumour growth and blood vessel formation [33], while the administration of Kringle 5 (K5) of human plasminogen, a potent angiogenesis inhibitor, by means of a recombinant AAV vector led to the inhibition of VEGF and tumour angiogenesis [34]. Lastly, the use of AAVrh10.BevMab, a rhesus serotype 10 adeno-associated vector coding for bevacizumab, demonstrated a significant reduction in tumour growth, reduced angiogenesis, and increased survival in mice with ovarian cancer [35]. 

Regarding the oncolytic virotherapy strategy, apart from HSV, the Vesicular Stomatitis Virus (VSV), and MeV described below, adenoviruses have also been employed and led to significant anti-tumour effects. Specifically, Han et al. designed the novel oncolytic Ad-M4 targeting signal transducer and activator of transcription 3 (*STAT3)* in ovarian cancer and showed that the aberrant expression and constitutive activation of STAT3 were key factors in cisplatin resistance in both ovarian cancer cell lines and ovarian cancer tissues samples [43]. The authors showed significant and selective inhibition of ovarian cancer cell survival and cisplatin-induced apoptosis both in vitro and in vivo following Ad-M4 administration [43]. In a similar approach, Wang et al. constructed the manganese superoxide dismutase-armed oncolytic Ad ZD55-MnSOD and used it together with cisplatin, achieving enhanced cisplatin-induced growth suppression and apoptosis in ovarian cancer cells lines and ovarian tumour xenografts [44]. 

Furthermore, and regarding the mutational compensation approach, most studies utilising Ads target either the mutant p53, a protein with a variety of anti-cancer functions in gynaecological cancers [78], or key apoptosis pathway regulators, such as Bim and Bid. In the former approach, which made its way to Phase II/III clinical trials, the replacement of the altered *p53* gene leads to significant anti-tumour effects [37,38]. Specifically, the in vitro and in vivo application of Ad-p53 resulted in the activation of apoptosis and taxol-sensitisation in ovarian cancer cells [38], while Gendicine, a gene therapy product approved for clinical use by the Chinese FDA in 2003, is now demonstrating very promising results, leading to a 90% patient response rate and 100% peritoneal effusion resolution [37]. In a recent study, Dai et al. [36] constructed two tumour-specific oncolytic adenoviruses coding for *Bim* (Ad-Bim) or truncated *Bid* (Ad-tBid) and performed gain-of-function assays in nine ovarian cancer cell lines. The authors observed increased cisplatin sensitisation and apoptosis following treatment with Ad-tBid in vitro and ex vivo, while following Ad-tBid intraperitoneal administration in mice with peritoneal disseminated ovarian cancer, they observed enhanced cisplatin-mediated anti-tumour effects [36]. Lastly, in regard to the immunopotentiation approach, HER2/neu, a protein shown to be overexpressed in ovarian cancer [79], was delivered to dendritic cells (DCs) by an AAV and primed these cells to express a self-tumour antigen. The above DC-loading elicited a strong MHC class I antigen presentation and led to a potent cytotoxic T lymphocyte response against ovarian cancer cells [46]. However, in view of the fact that the frequency of *HER2* overexpression in ovarian cancer is low [80], the above approach may have limited applicability. 

##### Herpes Simplex Virus (HSV), Measles Virus (MeV), and Vesicular Stomatitis Virus (VSV)

They comprise the main viral systems utilised in oncolytic virotherapy/suicide gene therapy approaches for ovarian cancer gene therapy. More specifically, Hartkopf et al. [45] designed a recombinant strain of MeV that carried a fusion cytosine deaminase (CD) protein, which enhanced the chemosensitivity of ovarian cancer cells in 5-fluoroucacil (5-FU) and used it in vitro and in vivo investigations. The authors demonstrated the effective infection and lysis of both human ovarian cancer cell lines and primary tumours [45]. In another study, Hanauer et al. [39], using a bispecific oncolytic MeV, with both HER2 and EpCAM as target receptors, demonstrated a significantly enhanced lysis of tumour cells in xenograft mice, highlighting the superiority of bispecific against monospecific oncolytic viruses [39]. Regarding HSV viruses, Goshima et al. [41] and Thomas et al. [40], in two independent studies, employed HSV in combination with immunostimulatory cytokines, such as GM-CSF and IL-12, respectively. The intraperitoneal injection (IP) of HSV-HF10 in a murine ovarian cancer murine model, together with the anti-tumour effect of GM-CSF, led to a reduction in tumour size and an increase in anti-tumour immune response [41]. Similarly, the intraperitoneal administration of HSV engineered to express IL-12 led to cytotoxic effects, reduced metastasis, and increased CD8^+^ T cell response [40]. Lastly, the use of VSV-GP, a potent oncolytic virus pseudotyped with the lymphocytic choriomeningitis virus (LCMV) envelope glycoprotein, together with the Jak1 and Jak2 inhibitor Ruxolitinib, led to transient tumour remission in mice following IP administration [42].

#### 3.1.2. Non-Viral Systems 

Strategies of this category utilise DNA/RNA plasmids to alter gene expression via transient transfection or nanoparticles to target a molecule expressed on the surface of cancer cells (Figure 3, Table 1).

##### Plasmids 

The use of these approaches has yielded therapeutic effects through suicide gene therapy, immunopotentiation, and mutation compensation strategies. More specifically, Sher et al. [50] constructed the hEndoyCD fusion protein, composed of the antiangiogenic endostatin and the *Escherichia coli* cytosine deaminase (CD) domain which converts 5-fluorocytosine (5-FC) to 5-FU, and delivered it to ovarian cancer cells via a plasmid vector (SV-hEndoyCD). The authors reported tumour-specific growth inhibition and increased survival in xenograft mouse models [50]. Regarding immunopotentiation approaches, two reports provide evidence of survival improvement [57,58]. Specifically, Hu et al. employed human umbilical cord CD34^+^ stem cells transfected with the pIRES2-IL-21-EGFP plasmid, carrying the anti-tumour cytokine IL-21, and demonstrated a therapeutic effect in ovarian cancer xenografts, possibly due to tumour-specific NK cytotoxicity, as a result of elevated levels of IFN-γ and TNF-α. Despite the gradual decrease in IL-21 in mice tumour tissues, overall extended survival was observed in these mice [57]. Similarly, Fewell et al. employed a pmIL-12/PPC vehicle carrying the anti-cancer IL-12 cytokine and demonstrated an efficient treatment for disseminated ovarian cancer as a result of the significant VEGF decrease and IL-12 and IFN-γ increase following IP administration [58]. 

Regarding mutation compensation, a plethora of studies have demonstrated significant anti-tumour effects following this approach [60,61,62,63,64,65,67,68,69,70]. Specifically, Wu et al., demonstrated a reversal of the malignant phenotype in vitro following the transfection of A2780 epithelial ovarian cancer (EOC) cells with a plasmid carrying the wild-type phosphatase and tensin homolog (*PTEN)* gene [60]. The above cells showed apoptosis induction, G1 arrest, and a decrease in migration and invasion [60]. In a different study, transfection with pcDNA3.1-p16/pcDNA3.1-vi5-hisA-eEF1A2 plasmids led to eEF1A2 down-regulation through the increase in tumour-suppressor *p16* and in turn inhibited cancer cell growth [61]. In a similar approach, Lu et al. [62] demonstrated a significant suppression of ovarian cell proliferation and migration in vitro and in vivo by the up-regulation of p16. The tumour-suppressing effects of the WW domain containing the oxidoreductase *(WWOX)* gene have been employed in targeted mutation compensation approaches in ovarian cancer, leading to significant results. Yan et al. [63] used a recombinant plasmid to transfect ovarian cancer stem cells and showed decreased proliferation, cell cycle arrest during the G0/G1 phase, and the induction of apoptosis through the up-regulation of caspase-3. A similar result was observed by Xiong et al. [65], employing WWOX delivery in liposomes, as described below. Recently Zhao et al. [64] showed that *WWOX* activation might inhibit chemoresistance and cell death induction in PTX-treated EOC cells. Specifically, the overexpression of WWOX in PTX-sensitive EOC cells using the pcDNA3.1-WWOX plasmid demonstrated the induction of apoptosis and the inhibition of autophagy and cell proliferation following PTX treatment [64]. The importance of the down-regulation of metastasis-associated in colon cancer 1 *(MAAC1),* metastasis-associated 1 *(MTA1),* and cyclooxygenase 2 *(COC2)* genes in ovarian cancer induction and progression was demonstrated by several studies, employing the down-regulation of their expression by shRNA/siRNA strategies [67,68,69,70]. Specifically, Zhang et al. used an shRNA plasmid to knock-down *MACC1* in ovarian carcinoma (OVCAR)-3 cells to demonstrate the inhibition of proliferation, migration, invasion, and induction of apoptosis both in vitro and in vivo in mouse models [67], while the siRNA interference strategy to knock-down *MTA1* in ovarian cancer cell line A2780 resulted in reduced migration, invasion, and adhesion and increased apoptosis in vitro [68]. Finally, the knock-down of cyclooxygenase 2 (*COX2*) by two independent groups [69,70] highlighted the role of the *COX2* gene in ovarian cancer cell proliferation, invasion, migration, and growth. 

##### Nanoparticles (NPs)

The use of these systems, primarily in suicide gene therapy and mutation compensation targeted approaches against ovarian cancer, has also yielded significant results. Specifically, Bai et al. transferred gelonin toxin in ovarian cancer cells using cationic heparin PEI (HPEI) nanogels and managed to reduce cancer cell growth and induce apoptosis [51]. Furthermore, Huang et al. [52] delivered diphtheria toxin subunit-A (DT-A) DNA in mice with ovarian tumours by means of cationic polymer administered IP, placing it under the control of a human epididymis protein 4 (HE4) promoter, whose activity is increased in ovarian cancer cells. The latter promoter was also used to drive the *TK* gene, leading to the inhibition of tumour growth and increased survival in mice upon delivery [52]. In a similar approach, Cocco et al. [53] employed the IP administration of NPs engineered to deliver a plasmid encoding for DT-A and succeeded in inhibiting ovarian tumour growth. Recently, Sun et al. [54] managed to successfully deliver a GCV-converted HSV-*tTK* in ovarian cancer cells employing Tat/pDNA/C16TAB (T-P-C) NPs, demonstrating targeted delivery in mouse models with human ovarian cancer. 

Regarding the mutation compensation approach using nanoparticles, this involves the delivery of siRNA in NPs, targeting *EGFR* [66], the receptor of *Clostridium perfringens* enterotoxin claudin-3 *(CLDN3*) [74,75] and hypoxia-inducible factor 1-alpha *(HIF1-a*) [76]. To this end, Dickerson et al. knocked down *EGFR*, an oncogene highly expressed in ovarian cancer cells and implicated in cell proliferation, migration, and differentiation, using siRNA delivered in nanogels and showed increased sensitivity of these cells to docetaxel [66]. Similarly, the knock-down of *CLDN3* by siRNA [74] or shRNA [75] led to the inhibition of tumour growth, a reduction in cell proliferation and angiogenesis, and the induction of apoptosis. Lastly, the silencing of *HIF1-a*, often overexpressed in ovarian cancer by means of siRNA in NPs, led to the effective inhibition of cell proliferation [76]. Regarding lipoplexes, dendrimers, and liposomes, a significant number of studies [55,56,59,71,72,73,77,81] have reported efficient delivery in ovarian cancer cells, highlighting the significant contribution of NPs in targeted gene therapy applications. Concerning the suicide gene therapy approach, He et al. [55] used FRα-targeted folate-modified lipoplex loaded with an hTERT promoter-regulated plasmid encoding VSV matrix protein (MP) (F-LP/pMP) and transfected SKOV3 ovarian cancer cells. The F-LP/pMP treatment resulted in the significant inhibition of tumour growth, as demonstrated by the inhibition of cell proliferation, apoptosis induction, and the suppression of tumour angiogenesis, as well as by the extended survival of mice in a SKOV-3 tumour model [55]. Moreover, Kobayashi et al. [56], employing a PAMAM dendrimer to deliver carbonyl reductase 1 *(CRB1*), which reduces micro-vessel density and leads to apoptosis, observed increased survival in mice and the decreased proliferation and dissemination of cancer cells. 

Furthermore, in a study by Yang et al. [59], the delivery of the human pro-apoptotic gene *hPNAS-4* into ovarian cancer cells using cationic liposomes led to the efficient inhibition of tumour growth, the induction of apoptosis, and the inhibition of angiogenesis both in vitro and in vivo. Furthermore, the inhibition of the Wilms tumour 1 *(WT1*) gene, overexpressed in ovarian carcinoma but not in normal tissue, by an antisense oligodeoxynucleotide (ASODN) delivered in liposomes, led to the inhibition of ovarian cancer cell proliferation, cell cycle arrest, and increased apoptosis in vitro [71]. The delivery of shRNA against STAT3 in liposomes resulted in a reduction in the growth of intraperitoneal ovarian cancer through the inhibition of cell proliferation and apoptosis induction [72], while the delivery of siRNA against NOTCH1 in cationic cholesterol derivative-based liposomes led to the inhibition of growth and apoptosis induction in vitro [73]. As mentioned above, Xiong et al. [65] delivered WWOX in ovarian cancer cells by means of liposomes and documented decreased cancer cell growth and apoptosis induction. Lastly, the utilisation of the CRISPR/Cas9 system in a folate receptor-targeted cationic liposome (F-LP) to target the DNA methyltransferase 1 *(gDMNMT1)* gene, whose overexpression results in inactivation or tumour suppressor genes and increased ovarian cancer cell resistance, resulted in the inhibition of ovarian cancer tumours both in vitro and in vivo [77].

##### miRNAs

Large-scale microarray analysis has highlighted the role of many microRNAs [miR(s)] in different types of cancer, including ovarian and cervical cancer [82]. miRNAs in ovarian cancer have shown to have either a tumour-promoting or tumour-suppressing role, depending on whether their expression is up-regulated or down-regulated, respectively, and hence can be employed both as therapeutic targets, as well as biomarkers for diagnosis and/or prognosis [83]. Regarding ovarian cancer, Iorio et al. [84] performed an initial and comprehensive miRNAs comparison between normal and ovarian cancer tissues and showed that miR-14, mir-199a, miR-200a, miR-200b, and miR-200c were up-regulated in ovarian cancer and thus acted as oncogenic miRs (oncomiRs), while miR-15, miR-16, mir-140, mir-145, mir-199a, and miR-125b1 were down-regulated, pointing toward a tumour-suppressing role (tumour suppressor miRs). Further studies highlighted novel players, such as miR-214, miR-150, miR-140-5P, miR-21, miR-29a, and let-7a and provided more insights into the role of different miRNAs in ovarian cancer. 

More specifically, Yang et al. [85] showed that miR-214 and miR-150 are highly up-regulated in ovarian cancer and act as oncomiRs, inhibiting the expression of *PTEN* tumour suppressor gene, thus initiating the protein kinase B (ACT) pathway. On the other hand, the prevention of miR-PTEN interaction can inhibit ovarian cancer, primarily through the inhibition of cancer cell proliferation and chemotherapeutic drug resistance. Therefore, the down-regulation of both miR-214 and miR-150 expression could potentially have a therapeutic effect. Similarly, experiments by Lan et al. [86] showed that miR-140-5p expression is significantly decreased in ovarian cancer patients, while when up-regulated, it leads to the induction of ovarian cancer cell proliferation and migration. The authors also observed that miR-140-5p down-regulation could reduce sensitivity to cisplatin, and therefore, the up-regulation of the specific miRNA could lead to both the inhibition of proliferation and migration and an enhancement of cisplatin sensitivity. Regarding miR-21, Echevarria-Vargas et al. [87] showed that miR-21 is also associated with cisplatin resistance among ovarian cancer cells. Its expression was found to be up-regulated in cisplatin-resistant ovarian cancer cells, and therefore targeting miR-21 can lead to reduced tumour characteristics in the specific cells. The down-regulation of miR-29a in ovarian cancer tissues is associated with apoptosis [88] while restoring let-7A-3/let-b expression leads to a reduction in tumour growth both in vitro and in vivo and the inhibition of invasion and metastasis [89]. Furthermore, increased let-7a is associated with increased survival rates in patients receiving platinum-based chemotherapy [90]. Similarly, the up-regulation of miR-200 family members is associated with decreased metastasis, primarily through reduced epithelial–mesenchymal transition (EMT) and tumour angiogenesis. Pecot et al. [91] reported that the miR-200 family inhibits blood vessel formation by targeting IL-8 and CXCL-1; therefore, the expression of the miR-200 family to the tumour epithelium results in decreased tumour cell metastasis and angiogenesis. Moreover, the observation that miR-183 and miR-22 expression was elevated in the low, compared to the high form of metastatic cancer, coupled with the fact that increasing the expression of miR-183 and miR-22 resulted in EZRIN reduction, led to the conclusion that EZRIN inactivation through miR-183 and miR-22 can inhibit ovarian cancer metastasis [92]. Lastly, Aqeilan et al. [93] demonstrated that miR-15 and miR-16 are involved in the multi-drug resistance of ovarian cancer cells by acting on Bcl-2, while Luo et al. [94] reported the tumour suppressing role of miR-126 in cancer SKOV3 cells, by demonstrating a miR-126-mediated P21 (RAC1) activated kinase 4 (PAK4) down-regulation.

### 3.2. Cervical Cancer

Cervical cancer remains the fourth most common and most lethal cancer type in women, despite regular screening and prevention strategies [95]. Targeted gene therapy presents a promising approach for the treatment of the specific malignancy and focuses primarily on mutation compensation strategies, suicide gene therapy, oncolytic virotherapy, antiangiogenic strategies, immunopotentiation, and drug resistance therapies [3], employing both viral and non-viral systems.

#### 3.2.1. Viral Vectors

**Figure 4:**
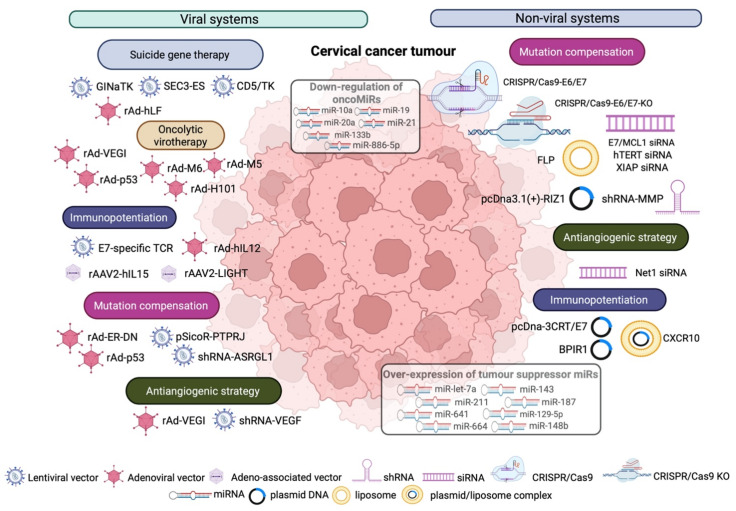
Gene therapy strategies for cervical cancer. They employ viral vectors, primarily adenoviral and adeno-associated viral vectors and lentiviral vectors, and non-viral systems, such as DNA plasmids, nanoparticles, CRISPR/CaS9, and shRNA/siRNA/miRNA approaches.

The main viral vectors used for the gene therapy of cervical cancer are LVs, Ads, and AAVs (Figure 4, Table 2).

##### Lentiviruses

Cervical cancer gene therapy approaches employ LVs primarily for mutation compensation, antiangiogenic, and suicide gene therapy strategies. The restoration of important tumour suppressor genes’ expression, such as tyrosine phosphatase receptor J *(PTPRJ),* asparaginase and isoaspartyl peptidase 1 *(ASRGL1),* and homeobox-containing 1 *(HMBOX1)*, has been successfully achieved with LVs. Specifically, Yan et al. [96] used pSicoR-PTPRJ LV to overexpress *PTPRJ,* a tumour suppressor gene whose expression is down-regulated in human cervical cancer tissues and demonstrated significant suppression of cell viability, migration, and growth in HPV-negative C33A cells. On the contrary, the knock-down of PTPRJ expression in the above cervical cancer cell line led to increased resistance to 5-FU-mediated apoptosis, verifying the importance of elevated PTPRJ expression for cervical cancer prevention. Moreover, Zhou et al. used a lentiviral shRNA to knock-down HMBOX1 expression in HeLa and C33A cancer cells and demonstrated increased radiosensitivity as a result of telomere shortening [97]. Lastly, the knock-down of ASRGL1 expression in SiHa cells by means of shRNA led to decreased proliferation, possibly through the reduced expression of CDK2 and cyclin A2 and the induction of apoptosis [98], which was characterised by the increased expression of the pro-apoptotic Bax and the decreased expression of anti-apoptotic Bcl-2. Regarding the antiangiogenic approach, Qi et al. used a lentiviral shRNA-VEGF construct to knock down VEGF expression in vitro and in vivo in nude mice and was able to inhibit tumour growth and tumour radiosensitivity [99]. One of the most promising approaches for cervical cancer gene therapy using lentiviruses is the genetic modification of T-cell receptors (TCRs) to target tumour-specific antigens. Based on the above, Jin et al. developed E7-specific T cells and achieved regression of HPV-positive mouse tumours [100]. The specific approach is currently under clinical trial NCT02379520 and is being used on patients with metastatic cervical cancer [101]. Regarding suicide gene therapy, the LV-mediated targeting of IAA and CD5 has been employed, while the GlNaTK retroviral vector was used to transfer the suicide *TK* gene into tumour cells. More specifically, Zhang et al. combined SEC3-ES LV targeting IAA and cisplatin and showed cell cycle arrest at the S phase, as well as the induction of apoptosis [102]. Similarly, Hao et al. studied the synergistic effect of two suicide gene systems, such as *CD/5-FC* and *HSV-TK/ganciclovir* (GCV), and demonstrated the inhibition of proliferation and enhanced apoptosis in vitro following lentiviral delivery combined with ultrasonic radiation [103]. Lastly, Chen et al. employed the GlNaTK retroviral vector to mediate *TK* transfer, which led to the inhibition of cell growth, increased apoptosis, and tumour radiosensitisation to cobalt-60 following GCV administration [104].

**Table 2:** Targeted gene therapy approaches for cervical cancer.

Gene Therapy Strategy	Gene	Delivery System	Outcome	Model/Species	References
Viral vectors					
Mutation compensation	*p53*	Adenoviral vector rAd-p53	Enhanced growth inhibition and apoptosis; increased 5-year overall survival rate	in vitro *(*human) in vivo *(*human)	[38,105]
Mutation compensation	*E6*	Adenovirus +siRNA	Successful p53 transduction following adenovirus treatment	in vitro *(*human)	[106]
Mutation compensation	*E6/E7*	Adenoviral vector Ad-ER-DN	Block of cell proliferation and induction of apoptosis	in vitro *(*human)	[107,108]
Suicide gene therapy	*hLF* (*human* *latoferin*)	Adenoviral vector rAd-hLF	Inhibition of tumour growth due to cell cycle arrest in G2/M phase and prolonged survival in xenograft models	in vitro *(*human) in vivo (mouse)	[109]
Oncolytic virotherapy	*E2*	Adenovirus rAd-M5	Growth suspension and increased apoptosis	in vitro *(*human) in vivo (mouse)	[110]
Oncolytic virotherapy	E6/E7	Adenovirus rAd-M6	Anti-tumour effect	in vitro (human) in vivo (mouse)	[111]
Oncolytic virotherapy	*Egr-1* *TRAIL*	Adenovirus rAd-pE3-Erg/TRAIL	Increased apoptosis and reduction in tumour size	in vitro *(*human) in vivo (mouse)	[112]
Oncolytic virotherapy	*E6*	Adenovirus rAd-H101	Down-regulation of HPV 16 E6 expression and increased p53 expression Enhancement of radiation anti-tumour efficiency	in vitro *(*human)	[113]
Oncolytic virotherapy/ Antiangiogenic strategy	*VEGF* *inhibitor* *(VEGI- 251)*	Adenoviral vector VEGI-armed	Inhibition of tumour neovascularization and induction of apoptosis	in vitro (human in vivo (mouse)	[114]
Immunopotentiation	*IL-12*	Adenoviral vector rAd-hIL12	Anti-tumour effect, inhibition of tumour growth and increase in survival rates	in vitro (mouse) in vivo (mouse)	[115]
Immunopotentiation	*IL-15*	Adeno-associated vector rAAV2-hIL15	Inhibition of cell growth	in vitro *(*human) in vivo (mouse)	[116]
Immunopotentiation	*TNF-14* *(light)*	Adeno-associated vector rAAV2-LIGHT	Suppression of tumorigenesis in a murine cervical cancer model	in vitro *(*mice/hamsters) in vivo (mouse)	[117]
Mutation compensation	*HMBOX1*	shRNA lentiviral vector	Increased apoptosis rate, increased radiosensitivity	in vitro *(*human)	[97]
Mutation compensation	*ASRGL1*	shRNA lentiviral vector	Decreased proliferation and apoptosis induction	in vitro *(*human)	[98]
Mutation compensation	*PTPRJ*	Lentiviral vector pSicoR-PTPRJ	Overexpression of PTPRJ led to significant suppression of viability, migration, and growth of cancer cells; Knock-down of PTPRJ increased C33A resistance to 5-FU-induced apoptosis	in vitro *(*human)	[96]
Antiangiogenic strategy	*VEGF*	Lentiviral vector shRNA-VEGF	Inhibition of cancer cell growth	in vitro *(*human) in vivo (mouse)	[99]
Immunopotentiation	*E7-specific TCR*	Lentiviral vector	Regression of HPV-positive mouse tumours	in vitro *(*human in vivo (human/mouse)	[100] [101]
Suicide gene therapy	*CD5/TK*	Lentiviral vector	Inhibition of proliferation and enhanced apoptosis	in vitro *(*human)	[103]
Suicide gene therapy	*IAA*	Lentiviral vector SEC3-ES	Cell cycle arrest at S phase and apoptosis	in vitro *(*human)	[102]
Suicide gene therapy	*HSV-TK*	GINaTK retroviral vector	Cell growth inhibition, increased apoptosis, and tumour radiosensitisation to cobalt-60	in vitro *(*human) in vivo (mouse)	[104]
Mutation compensation	*c-MYC*	Sendai virus (SeV)	Decrease in c-Myc and significant anti-tumour effects and apoptosis induction	in vitro (human) in vivo (mouse)	[118]
**Non-viral systems**					
Mutation compensation	*HPV18 E6/E7*	CRISPR/Cas9—E6/E7-KO	Robust knockout of E6 and E7 in cervical cancer cells, increased apoptosis and tumour size reduction	in vitro (human) in vivo (mice)	[119]
Mutation compensation	*HPV16 E6/E7*	CRISPR/Cas9 -nanoliposomes	Significant reduction in tumour size	in vitro (human) in vivo (mouse)	[120]
Mutation compensation	*E6/E7*	CRISPR/Cas9, TALEN	Growth inhibition, decreased proliferation, and increased apoptosis of HPV-positive cervical cancer cells	in vitro (human) in vivo (human)	[121,122] [101]
Mutation compensation	*HPV16 E7*	Nanoparticles composed of non- viral PBAE546 and CRISPR/Cas9 recombinant plasmid	Inhibition of growth of xenograft tumours and reversal of the cervical epithelial malignant phenotype of HPV16 transgenic mice	in vivo (mouse)	[123]
Mutation compensation	*HPV16 E7*	CRISPR/Cas9	Therapeutic effect in K14-HPV16 mice	in vitro (human) in vivo (mouse)	[124]
Mutation compensation	*E6/E7 and MCL1*	siRNA delivery by PEG-lipoplexes	Successful and prolonged delivery of active siRNAs by lipoplexes for HPV-induced lesions	in vitro (human)	[125]
Mutation compensation	*p53*	Polyamidoamine derivative AP-PAMAM	Anti-proliferative effect, induction of cell-cycle arrest at S phase, suppression of cancer cell migration and invasion	in vitro (human)	[126]
Mutation compensation	*XIAP*	siRNA	Cleaved caspase-3 activation and apoptosis in tumour tissue	in vivo (mouse)	[127]
Mutation compensation	*hTERT*	siRNA	Targeting hTERT induces growth inhibition and radiosensitivity; Knock-down of hTERT inhibits cervical cancer growth; Knock-down of HMB0X1 increases the radiosensitivity of cervical cancer cells	in vitro *(*human) in vivo (mouse)	[97,128,129]
Mutation compensation	*PEDF*	FRα-targeted liposome (FLP)	Significant anti-tumour activity, as demonstrated by significant growth inhibition and suppression of adhesion, invasion, and cancer cell migration	in vitro (human) in vivo (mouse)	[130]
Mutation compensation	*RIZ1*	pcDna3.1(+)-RIZ1 plasmid	Up-regulation of RIZ1 leads to reduction in cell proliferation and increased apoptosis; in combination with radiotherapy, it leads to increased apoptosis and DNA damage	in vitro (human)	[131]
Mutation compensation	*MMP9* *PTX3*	shRNA	Knock-down of PTX3 inhibits cell migration and invasion	in vitro (human) in vivo (mice)	[132,133]
Antiangiogenic strategy	*Net1*	siRNA	Angiogenesis and tumour growth reduction through VEGF down-regulation	in vitro *(*human) in vivo (mouse)	[134]
Immunopotentiation	*CXCL10*	Liposome-encapsulated plasmid	Growth and angiogenesis inhibition, apoptosis induction, arrest at G1 phase	in vitro *(*human) in vivo (mouse)	[135,136]
Immunopotentiation	*E6/E7*	pcDNA-3CRT/E7	Potent CD8 T-cell response and enhanced anti-tumour effects and survival	in vivo *(*mouse/human)	[137] [101]
Immunopotentiation	*E6/E7*	pcDNA-3CRT/E7 + IL2	Potent anti-tumour CD8 T-cells	in vivo (mouse)	[138]
Chemoresistance	*hTERT*	hTERT27	Inhibition of cell proliferation and induction of apoptosis	in vitro *(*human)	[139]
Chemoresistance	*BR1P1*	BRIP1 recombinant plasmid	Enhanced anti-tumour activity of cisplatin, increased apoptosis and angiogenesis suppression	in vitro (human)	[140]

##### Adenoviruses (Ad) and Adeno-Associated Viruses (AAV)

They comprise the majority of viral vectors used for cervical cancer gene therapy, often utilised for tumour suppressor gene restoration or blocking of oncogenic expression. The *p53* gene, a key regulator of cell proliferation, apoptosis, and genetic stability [141], plays a fundamental role in most gynaecological cancers and has therefore been a major candidate for most targeted gene therapy approaches [78]. More specifically, since the *p53* gene is often inactivated by HPV E6 protein in most cervical cancers [142]; either its delivery or inhibition of the E6 protein could result in significant antitumour effects. Indeed, Su et al. employing Genidicine^®^, a gene therapy product approved in 2003 by the China Food and Drug Administration (CFDA) for head and neck cancer gene therapy [143], showed that the injection of a recombinant human adenovirus engineered to express wild-type *p53* gene (rArd-p53) in cervical cancer patients, may lead to an increased 5-year overall survival rate [105]. Moreover, Kajitani et al., using siRNA for E6 protein in HeLa cells, demonstrated successful *p53* transduction following adenovirus treatment [106]. When the above transduction in HeLa cells was combined with PTX, it led to enhanced growth inhibition and apoptosis, as demonstrated by Liu et al. [38]. A further approach in tumour repressor gene restoration strategy is the use of rAd-ER-DN, an adenovirus expressing a dominant-negative estrogen receptor gene *(DNER),* to regulate estrogen imbalance, often associated with cervical cancer development. The use of rAd-ER-DN alone [107] or in combination with cisplatin and PTX [108] led to the blocking of cell proliferation and the induction of apoptosis in Ca Ski cervical cancer cells, primarily through the decrease in E6 and E7 oncoprotein expression. Regarding the oncolytic virotherapy approach, the use of five different Ads, i.e., rAd-M5, rAd-M6, rAd-53 [112], rAd-VEGFI-251, and rAd-H101 [113], demonstrated significant anti-tumour effects both in vitro and in vivo and led to increased apoptosis of cancer cells. rAd-M5 expressed the HPV *E2* gene, which negatively regulated E6 and E7 expression and led to growth suspension and increased apoptosis [110], while rAd-M6 employing antisense HPV16 E6/E7 DNA inhibited the expression of E6 and E7 and induced apoptosis in vitro and in vivo [111]. In another study, Xiao et al. used an oncolytic adenovirus expressing the VEGF inhibitor VEGI-251 and demonstrated the induction of apoptosis and the inhibition of neovascularisation [114], while Wang et al., employing rAd-p53 carrying the promoter of early growth response (Egr-1), and the anti-apoptotic TNF-related apoptosis-inducing ligand (*TRAIL)* gene showed increased radiotherapy-induced apoptosis in cell lines and tumour size reduction in xenograft mice [112]. Recently, Duan et al., using rAd-H101 oncolytic adenovirus targeting the E6 gene demonstrated enhanced radiation anti-tumour activity, primarily in C33A cells [113]. As far as the suicide gene therapy approach is concerned, the use of an Ad carrying the human lactoferrin (hLf), a glycoprotein involved in solid tumour growth reduction, demonstrated the inhibition of tumour growth, due to cell cycle arrest in the G2/M phase in HeLa cells and a prolonged survival in mice with cervical cancer [109]. Lastly, immunopotentiation strategies in cervical cancer gene therapy show promising results and involve both adenoviruses and adeno-associated viruses. In particular, the delivery of hIL-12, a cytokine with potent anti-cancer activity via the adenoviral vector (rAd-hIL12), led to the inhibition of tumour growth and an increase in survival rates in murine models [115]. Similarly, the delivery of the anti-tumour hIL-15 cytokine using the rAAV2-hIL15 led to the inhibition of growth in HeLa cells in vitro and in vivo in murine models [116], while rAAV2-LIGHT, an AAV vector carrying *LIGHT*, a TNF ligand, succeeded in suppressing tumorigenesis in a murine cervical cancer model [117].

#### 3.2.2. Non-Viral Systems 

Strategies of this category utilise primarily NPs to target a molecule expressed on the surface of cancer cells, as well as DNA and RNA plasmids or miRNAs to alter gene expression via transient transfection (Figure 4, Table 2).

##### Nanoparticles (NPs)

Gene therapy strategies by means of nanoparticles focus primarily on mutation compensation and immunopotentiation strategies. Regarding the former approach, Liu et al. [144] developed polyethylene glycol-polylactic acid (PEG-PLA) NPs linked to folate and targeted cancer cells through the folate receptor α (FRα), a membrane-bound protein mediating folate uptake. Although the authors observed enhanced gene transfection efficiency, higher compared to naked DNA, and reduced cytotoxicity, the strategies targeting FRα receptors are hampered by its heterogeneous expression among cervical cancer patients [145]. A few years later, Yang et al. overexpressed FRα, previously shown to be highly expressed in cervical cancer, using an FRα-targeted liposome (FLP) to deliver a pigment epithelium-derived factor *(PEDF)* gene into HeLa cells and observed significant anti-tumour activity, as demonstrated by significant growth inhibition, the suppression of adhesion and invasion, and cancer cell migration in vitro [130]. Lechanteur et al. used PEG-lipoplexes to mediate siRNA delivery against E6 and E7 oncoproteins and the anti-apoptotic myeloid cell leukaemia 1 (*MCL1)* gene; this was followed by the successful and prolonged delivery of active siRNAs by PEGylated lipoplexes, suggesting that this approach can treat HPV-induced lesions in vitro and in vivo in murine models [125]. Moreover, Han et al. using an AP-PAMAM derivative, induced an anti-proliferative effect in vitro by suppressing cancer cell migration and invasion, as well as the induction of cell-cycle arrest at the S phase [126]. Recently, the use of CRISPR/Cas9 against the HPV16 *E6* and *E7* encoding genes delivered in nanoliposomes led to a significant reduction in tumour size in murine mouse models [120]. Nanoparticles, composed of non-viral vector PBAE546 and a CRISPR/Cas9 recombinant plasmid against HPV16 E7 protein, led to the inhibition of growth in xenograft tumours and the reversal of the cervical epithelial malignant phenotype of the HPV16 transgenic mice [123]. Finally, regarding the immunopotentiation approach, Wang et al. [135] and Yang et al. [136] employed a liposome-encapsulated plasmid encoding *CXCL10*, an important cytokine with a potential therapeutic role in cervical cancer; they demonstrated an inhibition of growth and angiogenesis, which was associated with induction of apoptosis, mediated by the modulation of E6 and E7 oncoproteins expression both in vitro and in vivo.

##### Plasmids

The use of different plasmids in cervical cancer gene therapy approaches focuses on mutational compensation, antiangiogenesis, immunopotentiation and chemoresistance. With regards to blocking oncogenic expression, Hu et al. employed the genome editing approach using both the CRISPR/Cas9 and transcription-activator-like nucleases *(TALEN)* systems to disrupt the HPV16 *E7* oncoprotein coding gene [122]. The data documented induction of apoptosis and growth inhibition in HPV16-positive human cancer cells. The latter approach is being studied in Phase I clinical trials for the treatment of CIN-1 patients with HPV16 and HPV18 infection [101]. Recently, two groups demonstrated the therapeutic effect of targeting HPV *E6* and *E7* genes. Ling et al., using the CRISPR/Cas9 approach to delete the HPV18 *E6* and *E7* genes, achieved robust knock-out of these proteins and increased apoptosis and tumour size reduction [119]. In an attempt to compare the efficiency of CRISPR/Cas9 with the established TALEN approach, Gao et al. employed the former system against the HPV16 *E7* gene and succeeded in reverting cervical carcinogenesis, both in vitro and in vivo [124]. Another tumour suppressor gene with clinical importance in cervical cancer gene therapy is the retinoblastoma protein zinc finger gene 1 *(RIZ1)* since it can induce apoptosis and cell cycle arrest. Cheng et al. demonstrated that overexpression of RIZ1 expression in HPV16-positive cervical cancer cells could lead to impaired cell proliferation and increased apoptosis [131]. When this overexpression is combined with radiotherapy, it leads to increased apoptosis and DNA damage in HeLa and SiHa cells [146]. Blocking oncogenic expression can also be mediated by plasmids, primarily via the siRNA and shRNA approaches. Important genes such as X-linked inhibitor of apoptosis (*XIAP),* matrix metalloproteinase *(MMP),* and telomerase reverse transcriptase *(hTERT)* were targeted using the above approaches with promising results. Specifically, Wang et al. employed an RNA interference approach to silence the anti-apoptotic *XIAP* and demonstrated cleaved caspase-3 activation and apoptosis in tumour tissues [127]. Similarly, targeting of *hTERT*, which is often upregulated in cervical cancer and implicated in tumour cell invasion and proliferation, resulted in induction of growth inhibition, along with enhanced radiosensitivity in vitro [128], reduced cell proliferation, migration and invasion of cancer cells in vitro, as well as in inhibition of cell growth and tumorigenesis in nude mice [129]. The direct knock-down of the MMP9 protein involved in metastasis [132] or the indirect targeting of PTX3 [133], a molecule necessary for MMP2 and MMP-9 expression using shRNA plasmids, led to a reduction in cell migration and invasion, both in vitro and in vivo. In addition to the use of siRNAs for mutational compensation, their use in antiangiogenic strategies has also been demonstrated. Specifically, Zhang et al. used RNA interference to knock-down *Net1*, shown to be overexpressed in cervical cancer tissues, and demonstrated decreased proliferation, migration, and angiogenesis in SiHa cells, as well as a reduction in angiogenesis and tumour growth in vivo, via VEGF down-regulation [134]. Regarding immunopotentiation, E6 and E7 oncoproteins have been the optimal targets for immunotherapy and DNA vaccine production. Sun et al. developed a DNA vaccine using a plasmid encoding calreticulin (CRT) and E7 protein and managed to evoke a potent CD8 T-cell response, which could ultimately lead to anti-tumour effects and survival [137]. The co-administration of DNA encoding IL-2 in tumour-bearing mice elicited a stronger CTL response and enhanced anti-tumour effects [138]. The above approach is under clinical trial NCT00988559 [101]. Lastly, the issue of chemoresistance is also addressed by gene therapy to induce sensitivity in cervical cancer cells to chemotherapy. Examples of such approaches with promising results include the up-regulation of hTERTC27 and BRACA1-interacting protein (BRIP1) expression. More specifically, Lin et al. demonstrated that the overexpression of hTERTC27 expression led to increased sensitivity in HeLa cells to 5-FU, followed by inhibition of cell proliferation and increased apoptosis, primarily through the down-regulation of anti-apoptotic Bcl-2 expression and the up-regulation of pro-apoptotic caspases 3 and 9 [69]. Similarly, Liu et al. found that the overexpression of *BRP1* leads to enhanced cisplatin-mediated anti-tumour activity, increased apoptosis, and suppression of angiogenesis in vitro [140].

##### miRNAs

miRNAs involved in cervical cancer induction and development are classified into oncogenic miRNAs (oncomiRs) and tumour suppressor miRNAs (tumour suppressor miRs) [147,148] (Figure 4), and therefore, their down-regulation or overexpression, respectively, can be curative, while their presence in patients’ serum is considered as an important tool for cancer diagnosis and prognosis. More specifically and regarding oncomiRs, the most important ones include miR-10a, miR-19, miR-20, miR-21, miR-133b, and miR-886-5p [147]. Long et al. showed that miR-10a suppresses cell adhesion molecule L1, such as *(CHL1),* and thus leads to enhancement of tumour growth, metastasis, and invasion [149], while miR-19 is overexpressed in cervical cancer, and its silencing in SiHa cells led to a reduction in the proliferation and induction of apoptosis, through the up-regulation of Bax and down-regulation of Bcl-2 expression [150]. MiR-20 is a positive regulator of tyrosine kinase non-receptor 2 *(TNKS2),* an oncogene involved in metastasis and invasion [151]. In contrast, miR-21, which is the most well-known oncogenic miRNA, acts as a negative regulator of the tumour suppression gene programmed cell death 4 *(PDCD4)*, which normally inhibits cell proliferation and induces apoptosis [152], thus promoting tumour growth. Silencing by means of siRNA in cervical cancer cell lines led to inhibition of cell proliferation and induction of cell death by autophagy and caspase 3/7-mediated apoptosis [153]. Moreover, through targeting and regulating *CCL20*, a gene involved in tumour differentiation and metastasis, miR-20 was implicated in cervical squamous carcinogenesis [152]. Lastly, miR-133b is implicated in malignant transformation, originating from ERK and AKT1 signalling pathways [154], while miR-886-5p is negatively regulating the expression of the apoptotic protein Bax, as its silencing in SiHa cells led to induction of Bax expression and sequentially to apoptosis [155]. Protective miRNAs with important tumour suppressor activity primarily include miR-143, miR-148b, miR-211, miR-187, miR-641, miR-664, miR-let-7a, and miR-129-5p. More specifically, Liu et al. showed that the overexpression of miR-143 in HeLa cells led to suppression of Bcl-2 expression and thus the inhibition of cell proliferation and the induction of apoptosis [156]. Similarly, the transfection of miR-187 in SiHa cells also led to the downregulation of Bcl-2 and, in turn, to a reduction in cell growth and increased apoptosis [157]. Mou et al. employing miR-148b overexpression in HeLa cells demonstrated induction of G1/S cell-cycle arrest and apoptosis, mediated by caspase-3 [158], while Liu et al. showed that the down-regulation of IAP expression in SiHa cells following miR-211 overexpression, led to the inhibition of cancer cell growth and apoptosis enhancement [159]. Furthermore, the overexpression of miR-641 [160] and mir-644 [161] led to the inhibition of cervical cancer cell proliferation, migration, and invasion, with a concomitant increase in apoptosis. Ultimately, the ectopic expression of miR-let-7a in SiHa and HeLa cells led to the inhibition of cancer cell proliferation, migration, invasion, and apoptosis induction [162], while miR-129-5p overexpression in HeLa cells resulted in the inhibition of cell proliferation, increased cell-cycle arrest at the G0/G1 phase, and promoted apoptosis [163].

### 3.3. Endometrial Cancer

Despite being usually curable following surgery, endometrial cancer still presents as one of the most common female reproductive tract cancer types [4]. Occasionally aggressive tumours, such as uterine papillary serous carcinomas (UPSC), are observed, with most of them demonstrating aberrant expression of p53 [164]. Gene therapy strategies toward endometrial cancer involve both viral and non-viral systems, with the former employing primarily adenoviral and retroviral vectors and the latter plasmid DNA/RNA. 

#### 3.3.1. Viral Vectors

Ramondetta et al. performed an adenovirus-mediated expression of *p53* or *p21* in a papillary serous endometrial carcinoma cell line and demonstrated growth inhibition and apoptotic cell death [165]. Similarly, Ural et al. performed an in vitro suicide gene therapy approach using *HSV-TK* and documented inhibition of endometrial cancer cell growth [166]. A similar approach, but with the use of the pNF-κB plasmid, along with the gonadotropin-releasing hormone receptor (GnRH-R) agonist triptorelin and the prodrug GCV, resulted in reduced cancer cell growth, both in vitro and in vivo in mice [167]. Recently, Xia et al. [168] employed next-generation sequencing (NGS) in four out of the twelve patients enrolled in the clinical trial using the Ad-p53 vector, Genidicine^®^ [105] and observed reduced p53 expression in the tumours of three patients, all carrying mutations in tumour protein *p53* CREB binding protein *(CREBBP),* cyclin-dependent kinase inhibitor 2A *(CDKN2A),* LYN proto-oncogene *(LYN)* and Janus kinase 2 *(JAK2)* genes. These data provide strong evidence that NGS can aid in the recruitment of suitable patients for Ad-53 uterine gene therapy. 

#### 3.3.2. Non-Viral Systems

Effective gene transfer with a significant inhibition of cancer cells in vitro was also achieved with non-viral approaches. Specifically, Maurice-Duelli et al. demonstrated that the transfer of *PTEN*, a frequently mutated gene in endometrial cancer [169], using PEI-photochemical irradiation could lead to a 44% inhibition in cancer cell growth [170]. Moreover, the role of certain miRNAs in endometrial cancer has gained a lot of ground, highlighting their potential diagnostic and therapeutic value. In a detailed review, Banno et al. [171] highlighted the role of several miRNAs differentially expressed in endometrial cancer. Specifically, miR-185, miR-106a, miR-181a, miR-210, miR-423, miR-107, miR-let7c, miR-205, miR-449, and miR-429 were found up-regulated, while miR-let7e, miR-221, miR-30c, miR-152, miR-193, miR-204, miR-99b and miR-193b were significantly down-regulated, suggesting a tumorigenic or tumour-suppressing activity, respectively [171]. miR-129-2 and miR-152 are involved in the development of endometrial cancer via DNA methylation; miR-125b, mir-30c, miR-200b/c, and miR-429 are related to cisplatin resistance, while miR-125b, miR-30c, miR-194 and miR-34b regulate proliferation, metastasis and invasion of endometrial cancer cells [171]. Similarly, in a recent systematic review, Donkers et al. [172] highlighted the importance of miR-205, miR-200 family (miR-200a, miR-200b, and miR-200c), miR-135b, miR-182, miR-183, and miR-223 in endometrial cancer prognosis [172]. As the above miRNAs are found to be up-regulated in most endometrial cancers, the down-regulation of their expression may have therapeutic outcomes. Regarding miRNAs, such as miR-137, miR-129-3p, and miR-410, whose expression is down-regulated in endometrial cancer, the authors observed little to no consensus [172], and therefore, their role in both predicting and treating endometrial cancer remains vague.

## 4. Gene Therapy for Benign Gynaecological Disorders

### 4.1. Uterine Leiomyomas or Fibroids 

**Figure 5:**
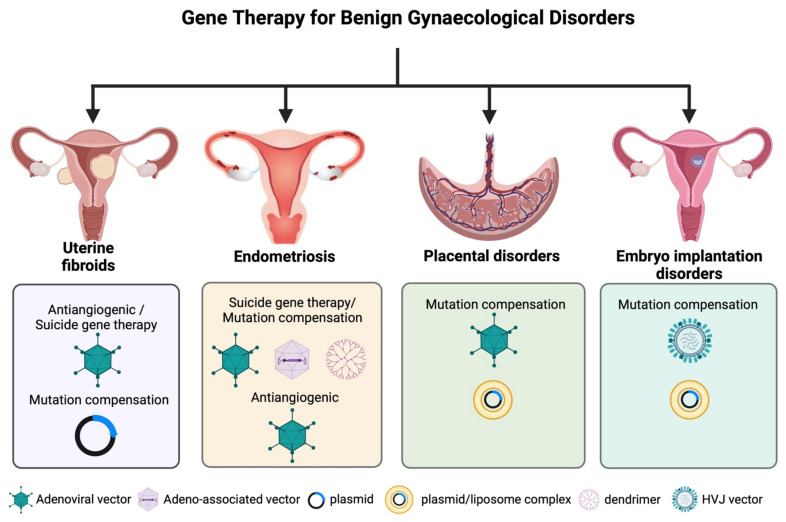
Gene therapy approaches toward benign gynaecological disorders. These include uterine fibroids, endometriosis, placental and embryo implantation disorders, using viral and non-viral systems. Successful approaches against uterine fibroids treatment employed primarily adenoviral vectors and plasmids, for endometriosis adenoviral vectors, adeno-associated vectors, and dendrimers, for placental disorders adenoviral vectors and plasmid/liposome complexes, while for embryo implantation disorders HVJ vectors and plasmid/liposomes complexes.

They are the most common benign tumours in reproductive-age women [173] and represent an attractive target for gene therapy, primarily due to their localised nature and slow growth rate [5,174]. The most common gene therapy approaches for fibroid treatment involve suicide gene therapy, antiangiogenic strategy, and mutation compensation and are summarised in Table 3 and Figure 5. The first attempt to employ gene therapy for uterine leiomyoma treatment came from Niu et al. 1998, who used a non-viral approach to deliver the suicide *TK* gene into leiomyoma cells [175]. Although the authors demonstrated significant cell death despite the low percentage of transfected leiomyoma cells, the above approach could only be applied in vitro. On the contrary, the use of improved Ads [174] with a leiomyoma-specific expression of critical therapeutic genes, such as *DNER* [176] and *HSV1-TK* [5], appear quite promising and present good candidates for human clinical trials. Furthermore, targeted and transduction-efficient Ads, such as Adenovirus-human somatostatin receptor subtype 2-arginine, glycine, and aspartate-thymidine kinase (Ad-SSTR-RGD-TK), given in combination with GCV, show promising results, both in vitro [177] and in vivo [178], as they lead to a significant reduction in proliferation and leiomyomas size, the induction of apoptosis, and the inhibition of angiogenic- and extracellular matrix-related genes. Recently, C-terminal Src kinase (*CSK2*) [179] and high mobility group AT-hook 2 *(HMGA2)* [180] were highlighted as key players in leiomyomas tumorigenesis. Specifically, *CSK2* was shown to be highly expressed in uterine leiomyosarcomas compared to leiomyomas, and its overexpression was associated with tumour progression and poor prognosis, while *CSK2* silencing using siRNA led to the inhibition of proliferation and cell cycle progression, decreased migration, invasion, and colony formation [179]. Additionally, the overexpression of *HMGA2* in leiomyomas was shown to be associated with increased vasculature density, demonstrated by the increased expression of angiogenic factors and receptors, such as VEGFA, EGF, bFGF, TGFα, VEGFR1, and VEGFR2, which likely contribute to tumour growth [180]. Its important role in angiogenesis appears to be through IGF2BP2-mediated pAKT activity [180].

### 4.2. Endometriosis 

It is a chronic, estrogen-dependent disease characterised by the presence of endometrial tissue outside the uterine cavity, often associated with subfertility [5,198]. In the early 2000s, the above benign gynaecological abnormality made its way to gene therapy applications (Table 3 and Figure 5). Specifically, Dabrosin et al. using a murine model of endometriosis and employing an adenovirus expressing the murine angiostatin gene managed to completely eradicate endometriotic lesions within two weeks [181]. A similar antiangiogenic strategy was followed by Sun et al., who used an AAV carrying endostatin (rAAv-endostatin-EGFP) and demonstrated the inhibition of angiogenesis in endometriotic lesions both in vitro and in vivo [182]. Similarly, but using an endostatin plasmid/PAMAM dendrimer instead, Wang et al. also demonstrated inhibition of endometriosis development following transfection [185]. Following the above, Othman and co-workers studied the effects of *DNER* gene transfer into endometriosis cells using an Ad-DNER vector and showed that it could induce significant cell death and reduce pro-inflammatory and angiogenic cytokine production by the specific cells [183]. Both of the above studies used an Ad5 viral vector; however, CRADs, such as Ad-SLP1-luc, Ad-hepanarase-luc [184], and Ad-sft-1 [186], achieved higher reporter gene expression and, therefore, may constitute better candidates for the gene therapy of endometriosis. In addition to antiangiogenic strategies, suicide gene therapy and mutation compensation have also been very efficient for endometriosis treatment. More specifically, the use of adenoviral vectors carrying the *TK* gene (Ad-TK) led to significant cell death induction and reduction in endometrial lesions in vivo [199]. Furthermore, regarding mutation compensation, Goncalves et al. employed Ad-p27, an adenovirus carrying *p27^kip1^* coding gene, shown to be down-regulated in women with endometriosis [200] and demonstrated cell cycle arrest and reduced proliferation [188,189]. Lastly, Paupoo et al., employing CRAd-S-pK7, an adenoviral vector with dual advantages of infection enhancement and promoter specificity, demonstrated high replication rates and cell-killing efficiencies in vitro [190].

### 4.3. Placental Disorders 

The potential contribution of gene therapy for placental abnormalities and functional restoration deficiencies was first introduced by Senut et al. [201] by injecting genetically modified cells into rat placenta, which demonstrated the increased secretion of gene products in fetal circulation. Furthermore, Xing et al. managed to successfully transfect rat placenta by either systemic administration or intra-placental needle injection of adenoviruses without altering the fetal genome [191]. However, as intra-placental needle injection may result in fetal-maternal barrier breakdown, Heikilla and co-workers performed a catheter-mediated intravascular gene transfer with adenoviruses, plasmid/liposomes and plasmid/PEI complexes and demonstrated that placental trophoblastic cells could be efficiently transfected with adenoviruses when delivered directly into uterine arteries [192]. On the contrary, and despite their ability to transfect fetal membranes and the basal plate more efficiently than adenoviruses, plasmid/PEI and plasmid/liposome complexes were not as efficient in transfecting placental trophoblastic cells [192]. Similarly, the Westwood group successfully used an adenovirus to deliver sense or antisense *IGF-I* or *IGF-II* cDNA into human primary placental fibroblasts (PPF), aiming to study the effects of overexpression or inhibition of these genes on placental cell proliferation, migration, and survival [193]. The authors reported that placenta cells could be efficiently transfected by adenoviruses, verifying the potential therapeutic outcome of gene therapy in placental disorders. Based on the above, Keswani et al. employed the adenoviral vector Ad-IGF-1 in vivo in mice, demonstrating the restoration of birth weight [194]. Similarly, Carr et al. showed that using Ad-VEGF led to an improvement in fetal growth and an increased fetal growth rate [195].

### 4.4. Embryo Implantation Disorders 

The proof of principle that genetic manipulation of the adult uterine endometrium has therapeutic potential against implantation disorders came from Bagot et al. in early 2000 [196]. The authors first reported that the alteration of maternal Hoxa10 expression, previously shown to be expressed in human endometrium during implantation [202] by in vivo gene transfection, affects implantation in mice [196]. Based on this finding, they overexpressed the maternal homeobox A10 (*Hoxa10)* gene using a pcDNA3.1(+)/HOXA10a, a liposome plasmid that constitutively expresses *Hoxa10*, and achieved a significant increase in litter size. All of the mice pups were born following a normal gestation of 17–20 days, were normal in size and exhibited no morphological abnormalities. Similarly, Nakamura et al. [197], employing the hemagglutinating virus of Japan envelope vector (HVJ), showed that NF-κB expression determines the timing of implantation via control of LIF expression and that manipulation of its expression may alter the implantation outcome. Microarray-based bioinformatics experiments by Horcajadas et al. [203] led to the identification of a huge number of transcripts regulated during implantation and set the basis for many in vivo gene transfer approaches. Furthermore, a multi-omics approach for the identification of successful implantation biomarkers [204] can be a useful tool for predicting a successful implantation outcome.

## 5. Clinical Trials

Despite the plethora of published data regarding the pre-clinical evaluation of viral and non-viral gene therapy approaches toward gynaecological cancers, only a small percentage has reached the clinical phase, with most of them not progressing beyond Phase I/II. According to clinicaltrials.gov [101] and using the terms “ovarian cancer”, “cervix cancer”, and “endometrial cancer” in combination with “gene therapy” as keywords, followed by manual exclusion of trials not involving gene therapy approaches, there are 18 clinical trials for ovarian gene therapy, 11 for cervical gene therapy, and three for endometrial gene therapy, with only a few currently recruiting patients. There are no clinical trials for gene therapy approaches against benign gynaecological disorders. Table 4 summarises the clinical trials for ovarian, cervical, and endometrial cancer gene therapy.

Regarding ovarian cancer gene therapy clinical trials, out of the 18 studies, three employed mutation compensation, three suicide gene therapies, and one oncolytic virotherapy strategy, while the remaining 11 used immunopotentiation approaches. In detail, in Phase I NCT00003450 and NCT00003588 clinical trials, Ads alone were used to deliver the *p53* gene, while in the Phase II NCT02435186 study, adenoviral-mediated *p53* delivery was combined with cisplatin and PTX chemotherapy. None of the above clinical trials have been posted or had their results published yet. With regards to suicide gene therapy approaches, clinical trials NCT00964756, NCT01997190, and NCT00005025 employed adenoviral or HSV vectors to deliver the *TK* gene in patients with recurrent ovarian cancer. Published data from Phase I clinical trial NCT00964756 using the Ad5-SSTR/TK-R-GD vector highlighted its safety and effectiveness, with five out of twelve patients showing stable disease and one out of five demonstrating complete disease resolution [205]. In the ongoing Phase I/II clinical trial NCT02068794, MSCs are used as carrier cells for oncolytic measles virus encoding thyroidal sodium iodide symporter (MeV-NIS) in patients with ovarian cancer. 

Regarding immunopotentiation approaches, in the Phase I clinical trial NCT00066404, adenoviral-mediated *IFN-β* transfer induced consistent anti-tumour immune responses; however, the rapid production of anti-adenoviral neutralizing antibodies following second dose administration led to minimized target cell transduction and IFN-β production [206]. To circumvent the above problem, the authors plan to administer a second dose as early as 3 days after the first one and combine adenoviral administration with chemotherapy or surgery. 

The rest clinical trials involve administration of engineered T cells expressing either neoantigen-specific TCRs (NCT03412877, NCT05194735, NCT03970382, NCT04102436) or anti-carcinoembryonic antigen (CEA) (NCT00004178), anti-mesothelin (NCT01583686) or folate binding protein (NCT00019136) chimeric TCRs. Specifically, for patients with solid tumours, there are two clinical trials (NCT02366546 and NCT02096614) assessing the in vivo kinetics and safety of TBI-1301 (NY-ESO-1-specific TCR) and TBI-1201 (MAGE A4-specific TCR), in combination with cyclophosphamide and fludarabine. All the above approaches employing engineered T cells have gained a lot of ground, leading to promising results toward cancer immunotherapy [207]. 

Overall, out of the 18 clinical trials described above, 16 employed viral gene delivery systems, while two, specifically the NCT04102436 and NCT00381173 trials, used non-viral approaches, such as the *Sleeping Beauty* transposon/transposase system or plasmid DNA, respectively. Specifically, in the latter study, the administration of ZYC300, a DNA plasmid encoding the tumour-associated carcinogen activator cytochrome P450 1B1 *(CYP1B1)* [208], was used to treat 17 patients with advanced-stage progressive cancer and led to CYP1B1 immunity induction and marked response to next regimen in six of them [209]. 

Regarding the cervical cancer clinical trials, eight out of ten clinical trials employ immunopotentiation approaches, while only two and specifically NCT03544723 and NCT03057912, are based on mutation compensation strategies. Clinical trial NCT03544723 is a Phase II multi-centre study to evaluate adenoviral-mediated *p53* delivery in combination with immune checkpoint inhibitors, such as anti-PD-1/anti-PD-L1 in patients with carcinomas approved for anti-PD-1 and anti-PD-L1 therapy. Accumulating evidence presented by Liu et al. shows that PD-1/PD-L1 inhibitors may also be a promising therapeutic approach for cervical cancer as well [210], and therefore the results of the above study (estimated completion date 31/12/2022) will contribute significantly toward efficient cervical cancer treatment. The NCT03057912 Phase I study assesses the safety and efficacy of the in vivo TALEN- and CRISPR/Cas9-mediated disruption of HPV16- and HPV18-E6/E7 oncoproteins. 

Regarding clinical trials employing immunopotentiation approaches, in the ongoing clinical trial NCT04180215, checkpoint inhibitors are administered in combination with vectors expressing HPV16+ antigen (HB-201 and HB-202) to assess their efficacy against patients with metastatic HPV16+ cancers, while clinical trial NCT00988559 evaluates the safety and efficacy of different routes of administration of pnGVL4a-CRT/E7 (detox) DNA vaccine in patients with HPV16+ cervical intraepithelial neoplasia (CIN)2/3. While the results of the former study will not become available before summer 2025, results of the latter clinical trial indicate induction of CIN2/3 remission following intramuscular or intralesional delivery of pnGVL4a-CRT/E7 (detox) vaccine. The other cervical cancer clinical trials involve the infusion of T cells engineered to express TCRs against HPV16/18 E6/E7 oncoproteins (NCT02379520 and NCT022280811), DP0401/0402 restricted MAGE-A3 tumour antigen (NCT02153905, NCT02111850), CEA (NCT00004178), or mesothelin (NCT01583686), usually in combination with cyclosphosphamide or fludarabine. The results of the above clinical trials are not published yet; however, the therapeutic potential of the specific approach is quite promising [207]. Lastly, the anti-tumour effect of *IFN-β* transfer was clearly demonstrated in the NCT00066404 clinical trial [206], and it is expected that the revised experimental design will lead to an even more significant therapeutic outcome.

Regarding endometrial cancer clinical trials, all three (NCT05194735, NTC00004178, NCT00066404) employ immunopotentiation approaches discussed above and involve either infusion of CAR T cells or adenoviral-mediated *IFN-β* transfer. 

## 6. Conclusions and Future Directions

The aim of this review was to summarise all the major advances regarding gene therapy for gynaecological malignant and benign disorders and highlight their therapeutic potential and their eventual clinical application. It is clear that targeted gene therapy approaches, such as mutation compensation, antiangiogenesis, immunopotentiation, suicide gene therapy, and oncolytic virotherapy, have yielded promising results, employing both viral and non-viral systems. However, most of these strategies either remain in the preclinical phase or show reduced effectiveness as monotherapies and usually require a combination of chemotherapy and radiotherapy to demonstrate a therapeutic outcome. Furthermore, gene therapy in combination with immunotherapy, e.g., targeting CTLA-4 and PD-1, or with the emerging therapeutic angiogenesis inhibitors, may result in increased effectiveness, especially for ovarian cancer in advanced stages, where still no effective therapies exist. Moreover, there is increasing evidence for the therapeutic potential of CAR T-cell technology also, and clinical trials are currently assessing its efficacy, primarily in ovarian cancer, by recruiting patients [101] (Table 4). The continuous development of safe and effective vectors, the discovery of novel diagnostic markers, such as miRNAs discussed above, or of new players, such as long non-coding RNAs (lncRNAs) [211] and circular RNAs (circRNAs) [212] and in combination with more clinical trials beyond Phase I/II are expected to lead to more effective and radical therapeutic outcomes.

## Figures and Tables

**Table 3:** Targeted gene therapy approaches for benign gynaecological abnormalities.

Gene Therapy Strategy	Gene	Delivery System	Outcome	Model/Species	References
Uterine leiomyomas or fibroids					
Suicide gene therapy	*HSV-TK*	Non-viral approach	Significant cell death of leiomyoma cells	in vitro (human/rat)	[175]
Suicide gene therapy	*HSV-TK*	Adenoviral vector Ad-HSV1TK/GCV	Significant reduction in uterine fibroids volume, increased apoptosis and inhibition of cell proliferation in Eker rats	in vitro (human) in vivo (rat)	[174]
Suicide gene therapy	*Somatostatin receptor subtype 2/* *HSV-TK*	Adenoviral vector Ad-SSTR-RGD-TK	Significant reduction in leiomyomas size and proliferation, induction of apoptosis and inhibition of angiogenic- and extracellular matrix-related genes	in vitro (human) in vivo (mouse)	[177,178]
Suicide gene therapy/ Antiangiogenic gene therapy	*DNER/* *HSV-TK*	Adenoviral vector Ad-DNER	Leiomyoma-selective expression, decreased apoptosis, and proliferation	in vitro (human/rat)	[176]
Mutation compensation	*CKS2*	Plasmid si-CKS2	Cell cycle arrest and inhibition of cell proliferation, colony formation, migration, and invasion	in vitro (human)	[179]
**Endometriosis**					
Antiangiogenic strategy	*Angiostatin*	Adenoviral vector	Eradication of endometriosis lesions	in vitro (mouse)	[181]
Antiangiogenic strategy	*COL18A1* *(endostatin)*	Adeno-associated vector rAAV-endostatin-EGFP	Inhibition of angiogenesis in endometriotic lesions	in vitro *(*human) in vivo (mouse)	[182]
Antiangiogenic strategy	*DNER*	Adenoviral vector Ad-DNER Ad5 vector	Significant cell death and reduction in pro-inflammatory and angiogenic cytokine production by endometriosis cells	in vitro *(*human)	[183,184]
Antiangiogenic strategy	*COL18A1* *(endostatin)*	Endostatin plasmid/PAMAM dendrimers	Inhibition of endometriosis development	in vitro *(*human) in vivo (mice)	[185]
Antiangiogenic strategy	*VEGF receptor 1*	Adenoviral vector Ad-sft-1	Transduction flexibility	in vitro *(*human) in vivo (mouse)	[186]
Antiangiogenic strategy	*HPSE* (*heparanase)* *SLP1*	Adenoviral vector Ad-heparanase-luc Ad-SLP1-luc	Ad-heparanase-luc vector demonstrated an “Endometriosis ON, liver OFF” phenotype	in vitro *(*human)	[184]
Suicide gene therapy	*HSV-TK*	Adenoviral vector Ad-TK	Induction of significant cell death and reduction in endometrial lesions in vivo	in vitro *(*human) in vivo *(*mouse)	[187]
Mutation compensation	*p27^kip1^*	Adenoviral vector Ad-p27	Cell cycle arrest and reduced proliferation	in vitro (human)	[188,189]
Mutation compensation	*VEGF promoter*	Adenoviral vector CRAd-S-pk7	Endometriosis killing effect	in vitro *(*human)	[190]
**Placental disorders**					
Mutation compensation		Adenoviral vector	Successful transfection of placenta cells	in vitro (human) in vivo (mouse/rabbit)	[191,192,193]
Mutation compensation	*IGF-1*	Adenoviral vector Ad-IGF-1	Restoration of birth weight	in vivo (rabbit)	[194]
Mutation compensation	*VEGF*	Adenoviral vector Ad-VEGF	Improvement of fetal growth, increased fetal growth velocity	in vivo (sheep)	[195]
Mutation compensation	*IGF-1/IGF-II*	Plasmid/liposomes	Importance of overexpression or inhibition on placental cell proliferation, migration and survival	in vitro (human)	[192]
**Embryo implantation disorders**					
Mutation compensation	*Hoxa10*	Plasmid/liposome	Significant increase in litter size	in vivo (mouse)	[196]
Mutation compensation	*NF-* *κ* *B*	HVJ vector	NF-κB expression determines the timing of implantation, via control of LIF expression	in vitro (pigs)	[197]

**Table 4:** Gene therapy clinical trials for ovarian, cervical and endometrial cancer available online clinicaltrials.gov (accessed on 29 May 2022) [101].

Therapeutic Strategy	Phase	Reference	Intervention	Date (First–Last Posted)	Recruiting Status
**Ovarian cancer gene therapy clinical trials**					
Mutation compensation	Phase I	NCT00003450	Delivery of *p53* gene using Ad5CMV-p53	5 December 2003– 2 February 2021	Completed
Mutation compensation	Phase I	NCT00003588	Delivery of *p53* gene using Ad-53	26 August 2004– 8 February 2013	Completed
Mutation compensation	Phase II	NCT02435186	Delivery of *p53* gene using Ad-53 + cisplatin and PTX	6 May 2015– 6 May 2015	Unknown
Oncolytic virotherapy	Phase I/II	NCT02068794	Administration of oncolytic Measles virus-infected MSCs	21 February 2014– 15 March 2022	Recruiting
Immunopotentiation	Phase I	NCT00019136	Transfer of anti-CD3 stimulated peripheral blood lymphocytes transduced with a folate binding protein chimeric TCR (MoV-gamma chimeric TCR)	27 January 2003– 29 April 2015	Completed
Immunopotentiation	Phase I	NCT00004178	Transfer of recombinant CEA immunoglobulin TCR (IgTCR)	19 April 2004– 10 June 2011	Completed
Immunopotentiation	Phase I/II	NCT01583686	Transduction of PBLs with retroviral vector and transfer of anti-mesothelin CAR TCR	24 April 2012– 14 October 2019	Terminated
Immunopotentiation	Phase I	NCT00066404	Delivery of human *IFN-β* gene using recombinant adenovirus Ad.IFN-β	7 August 2003– 13 May 2020	Completed
Immunopotentiation	Phase II	NCT03412877	Infusion of autologous T cells engineered to express neoantigens-reactive TCRs	29 January 2018– 27 May 2022	Recruiting
Immunopotentiation	Phase I/II	NCT05194735	Infusion of autologous T cells engineered to express neoantigens-reactive TCRs + IL-2	18 January 2022– 11 April 2022	Recruiting
Immunopotentiation	Phase II	NCT04102436	Infusion of autologous T cells engineered with *Sleeping Beauty* transposon/transposase system (non-viral approach) to express neoantigens-reactive TCRs	25 September 2019– 27 May 2022	Recruiting
Immunopotentiation	Phase I	NCT00381173	Administration of ZYC300 using plasmid DNA + cyclophosphamide	27 September 2006– 14 May 2013	Completed
Immunopotentiation	Phase 1a/1b	NCT03970382	Infusion of autologous T cells engineered to express neoantigen- reactive TCR + nivolumab	31 May 2019– 8 February 2022	Active, not recruiting
Immunopotentiation	Phase I	NCT02366546	Administration of TB1-1301 (NY-ESO-1 specific TCR) in solid tumours cyclophosphamide + fludarabine	19 February 2015– 24 October 2018	Active, not recruiting
Immunopotentiation	Phase I	NCT02096614	Administration of TB1-1201 (MAGE-A4-specific TCR) in solid tumours + cyclophosphamide + fludarabine	26 March 2014– 18 June 2021	Completed
Suicide gene therapy	Phase I	NCT00964756	Therapeutic gene delivery using Ad5.SSTR/TK.RGD + GCV	25 August 2009– 13 February 2013	Completed
Suicide gene therapy	Phase I	NCT01997190	Administration of AdV-tk + valacyclovir	28 November 2013– 4 December 2020	Completed
Suicide gene therapy	Phase II	NCT00005025	Administration of HSV-tK + GCV	5 May 2003– 6 November 2013	Unknown
**Cervical cancer gene therapy clinical trials**					
Mutation compensation	Phase II	NCT03544723	Delivery of *p53* gene using Ad-53 and anti-PD-1/anti-PD-L1	4 June 2018– 9 June 2020	Recruiting
Mutation compensation	Phase I	NCT03057912	Disruption of HPV16- and HPV18-E6/E7 oncoproteins by TALEN and CRISPR/Cas9 approaches	20 February 2017– 9 June 2017	Unknown
Immunopotentiation	Phase I/II	NCT04180215	Administration of HB-201 alone or in combination with HB-202 in HPV 16 + patients + checkpoint inhibitors	27 November 2019– 24 May 2022	Recruiting
Immunopotentiation	Pilot study	NCT00988559	Different routes of administration of DNA vaccine pnGVL4a-CRT/E7	2 October 2009– 9 July 2018	Completed
Immunopotentiation	Phase I	NCT02379520	Administration of HPV-16/18 E6/E7- specific T cells + fludarabine	5 March 2015– 11 January 2022	Active, not recruiting
Immunopotentiation	Phase I/II	NCT022280811	Infusion of T cells expressing an HPV E6-specific TCR + cyclophosphamide and IL-2	2 November 2014– 6 September 2017	Completed
Immunopotentiation	Phase I/II	NCT02153905	Infusion of T cells engineered to express an anti-MAGE A3 HLA-A*01-restricted TCR + fludarabine	3 June 2014– 17 June 2019	Terminated
Immunopotentiation	Phase I/II	NCT02111850	Transfer of anti-MAGE A3-DP4- restricted TCR + IL-2	11 April 2014– 29 March 2022	Completed
Immunopotentiation	Phase I	NCT00004178	Transfer of anti-CEA immunoglobulin TCR (IgTCRs)	19 April 2004– 10 June 2011	Completed
Immunopotentiation	Phase I	NCT00066404	Delivery of human *IFN-β* gene using recombinant adenovirus (Ad.IFN-β)	7 August 2003– 13 May 2020	Completed
Immunopotentiation	Phase I/II	NCT01583686	Administration of anti-mesothelin CAR TCR + fludarabine	24 April 2012– 14 October 2019	Completed
**Endometrial cancer gene therapy clinical trials**					
Immunopotentiation	Phase I/II	NCT05194735	Infusion of autologous T cells engineered to express neoantigens-reactive TCRs + IL-2	18 January 2022– 11 April 2022	Recruiting
Immunopotentiation	Phase I	NTC00004178	Transfer of recombinant anti-CEA immunoglobulin TCR (IgTCR)	19 April 2004– 10 June 2011	Completed
Immunopotentiation	Phase I	NCT00066404	Delivery of human *IFN-β* gene using recombinant adenovirus	7 August 2003– 13 May 2020	Completed
